# Unraveling the Transmission Ecology of Polio

**DOI:** 10.1371/journal.pbio.1002172

**Published:** 2015-06-19

**Authors:** Micaela Martinez-Bakker, Aaron A. King, Pejman Rohani

**Affiliations:** 1 Department of Ecology & Evolutionary Biology, University of Michigan, Ann Arbor, Michigan, United States of America; 2 Center for the Study of Complex Systems, University of Michigan, Ann Arbor, Michigan, United States of America; 3 Fogarty International Center, National Institutes of Health, Bethesda, Maryland, United States of America; The Pennsylvania State University, UNITED STATES

## Abstract

Sustained and coordinated vaccination efforts have brought polio eradication within reach. Anticipating the eradication of wild poliovirus (WPV) and the subsequent challenges in preventing its re-emergence, we look to the past to identify why polio rose to epidemic levels in the mid-20th century, and how WPV persisted over large geographic scales. We analyzed an extensive epidemiological dataset, spanning the 1930s to the 1950s and spatially replicated across each state in the United States, to glean insight into the drivers of polio’s historical expansion and the ecological mode of its persistence prior to vaccine introduction. We document a latitudinal gradient in polio’s seasonality. Additionally, we fitted and validated mechanistic transmission models to data from each US state independently. The fitted models revealed that: (1) polio persistence was the product of a dynamic mosaic of source and sink populations; (2) geographic heterogeneity of seasonal transmission conditions account for the latitudinal structure of polio epidemics; (3) contrary to the prevailing “disease of development” hypothesis, our analyses demonstrate that polio’s historical expansion was straightforwardly explained by demographic trends rather than improvements in sanitation and hygiene; and (4) the absence of clinical disease is not a reliable indicator of polio transmission, because widespread polio transmission was likely in the multiyear absence of clinical disease. As the world edges closer to global polio eradication and continues the strategic withdrawal of the Oral Polio Vaccine (OPV), the regular identification of, and rapid response to, these silent chains of transmission is of the utmost importance.

## Abstract 2

Using models to reveal the epidemiology of historic poliovirus transmission in the US, this study reconstructs the millions of silent infections and identifies why polio epidemics are explosive, seasonal, and vary geographically.

## Author Summary

Thanks to global vaccination efforts, poliovirus is on the brink of worldwide eradication. However, achieving eradication and preventing re-emergence requires intimate knowledge of how the virus persists. In order to understand a system that is complicated by heavy human intervention, such as vaccination, it is important to establish a baseline by studying that system in the absence of intervention. Historical epidemics that predate the use of vaccines can be used to disentangle the epidemiology of disease from vaccine effects. Using historical polio data from large-scale epidemics in the US, we fitted and simulated mathematical models to track poliovirus and to reconstruct the millions of unobserved subclinical infections that propagated the disease. We identified why polio epidemics are explosive and seasonal, and why they vary geographically. Our analyses show that the historical expansion of polio is straightforwardly explained by the demographic “baby boom” during the postwar period rather than improvements in hygiene. We were also able to demonstrate that poliovirus persisted primarily through symptomless individuals, and that in the event of local virus extinction, infection was reintroduced from other geographic locations.

## Introduction

Poliovirus, like other members of *Picornaviridae*, usually generates mildly symptomatic infection. However, the clinical manifestation of polio, Acute Flaccid Paralysis (AFP), can result when the virus invades the central nervous system [1]. Wild poliovirus (WPV) is transmitted fecal–orally and in the Northern Hemisphere exhibits seasonal epidemics in late summer and autumn [1–3]. Polio outbreaks continue today within this narrow seasonal window in Pakistan and Afghanistan [4,5], but the seasonal transmission structure of polio remains unexplored.

Propelled by public support, the race for the polio vaccine during the post-World War II era led to the development of the Inactivated Polio Vaccine (IPV) and the Oral Polio Vaccine (OPV), which reduced the global incidence to less than 0.1% of prevaccine levels [6]. Missing the 2014 goal of globally stopping WPV transmission has left eradication elusive, primarily because of political and social obstacles for effective vaccine distribution, including vaccine hesitancy and mistrust. In light of this—and the call for innovative solutions [7]—an understanding of polio’s ecology can help guide alternative strategies. Looking toward eradication and beyond, a polio-free world requires an understanding of the mode by which polio originally emerged and historically persisted. We contend that a retrospective study of the ecology of WPV in the absence of vaccine interventions can inform future planning and may pinpoint vulnerabilities in WPV’s epidemiology that could be leveraged for eradication.

Ironically, because of the success of polio vaccination, critical features of WPV transmission remain obscure. The low global incidence of polio (due to high vaccine coverage), in combination with the relative rarity of symptomatic infections, limits the amount of epidemiological data with which to study transmission. Furthermore, data limitations regarding vaccine coverage in developing countries confound transmission studies, making it difficult to disentangle the effects of the vaccines, demography, and transmission. Therefore, we took advantage of a dataset of unprecedented size and resolution in both space and time to gain insights into the drivers of polio’s historical expansion and the ecological mode of its persistence in the prevaccine period.

We present analyses of spatially-replicated incidence reports from the prevaccine era in the United States and built mechanistic transmission models that incorporate these data to reconstruct the unobservable infection dynamics. Our analyses allow us to dissect three axes of polio epidemiology: (i) geographical and seasonal variation in transmission, (ii) the role of demography in determining incidence, and (iii) the mode by which polio persists.

## Methods

### Data

**Fig 1:**
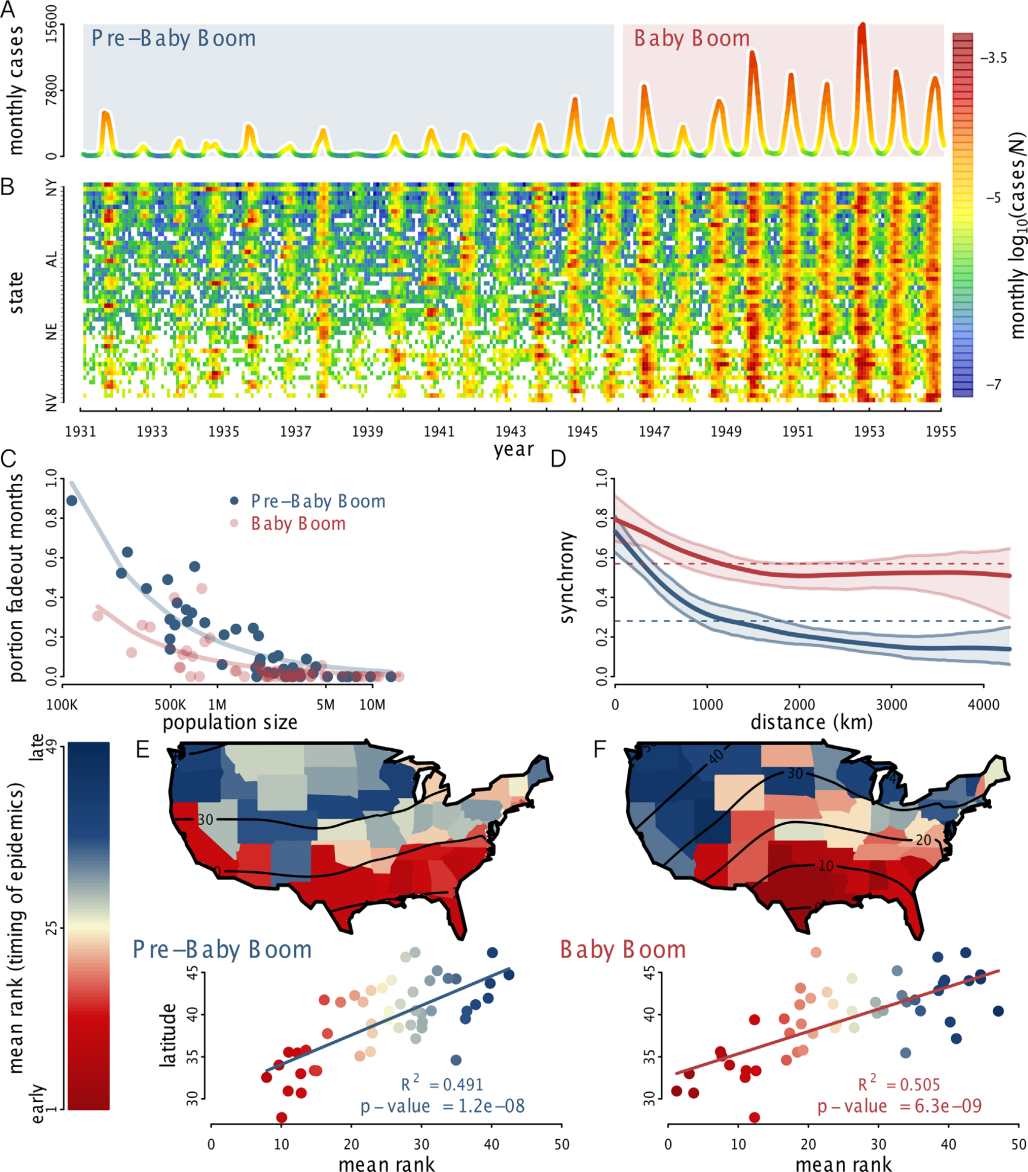
Spatiotemporal patterns in polio incidence. (A) Total monthly case reports, 1931–1954, color-coded by per capita incidence. (B) Log-transformed per capita incidence by state, ranked top-to-bottom by population size. (C) Disease fadeout frequency as a function of state population size, during the pre-baby boom and baby boom eras. The lines represent fitted negative exponential curves, which tended toward zero. (D) Pairwise epidemic synchrony between states during the pre-baby boom and the baby boom. Mean and 95% bootstrap confidence envelope shown. (E, F) Relative timing of polio epidemic peaks during the (E) pre-baby boom and (F) baby boom eras. Color indicates mean rank of each state across years; lower rank indicates earlier epidemic peak. Below each map, relative timing is regressed on latitude. Lower latitude states had significantly earlier epidemic peaks. The data used to make this figure can be found in S1 Data, S2 Data, and S3 Data.

We examined monthly polio case reports (January 1931–December 1954) from the US Public Health Service Morbidity and Mortality Weekly Reports as compiled by [8] and the CDC for each of the 48 contiguous US states and the District of Columbia (Fig 1A and 1B); data provided in the Supporting Information. Prior to 1945, the cases in these data were predominantly paralytic [1,3]; however, during the later period of this study, nonparalytic cases comprised more than 40% of reported cases in populous cities such as New York, Detroit, Kansas City, and Sacramento [9]. In addition to polio case data, we obtained numbers of births by state from 1931 onward from the US Vital Statistics and state population sizes from the Population Distribution Branch of the US Census Bureau. Data from Vital Statistics are housed in the CDC online repository: National Center for Health Statistics, Products, Vital Statistics. The Census Bureau data were obtained from their Population Estimates Repository, historical data pre-1980; data also provided in the Supporting Information. The polio dataset—with cases detailed weekly—has now been independently digitized and is freely available and maintained online through the University of Pittsburgh Project TYCHO. Birth data were not available for Texas and South Dakota beginning in 1931 but began in 1932 and 1933, respectively. For exploratory analyses, we quantified the relationship between disease fadeouts and population size. A threshold of 3 mo without a reported infection was chosen to define a fadeout [10]. The portion of fadeout months was taken as the ratio of fadeout months to total months in Fig 1C. To estimate spatial synchrony, we used the nonparametric spatial correlation function [11,12]. To measure the relative timing of polio epidemic peaks for each state and each year, the 1 yr wavelet band phase angle was computed [13] and used to rank states earliest to latest based on their epidemic peak timing.

### Models

**Fig 2:**
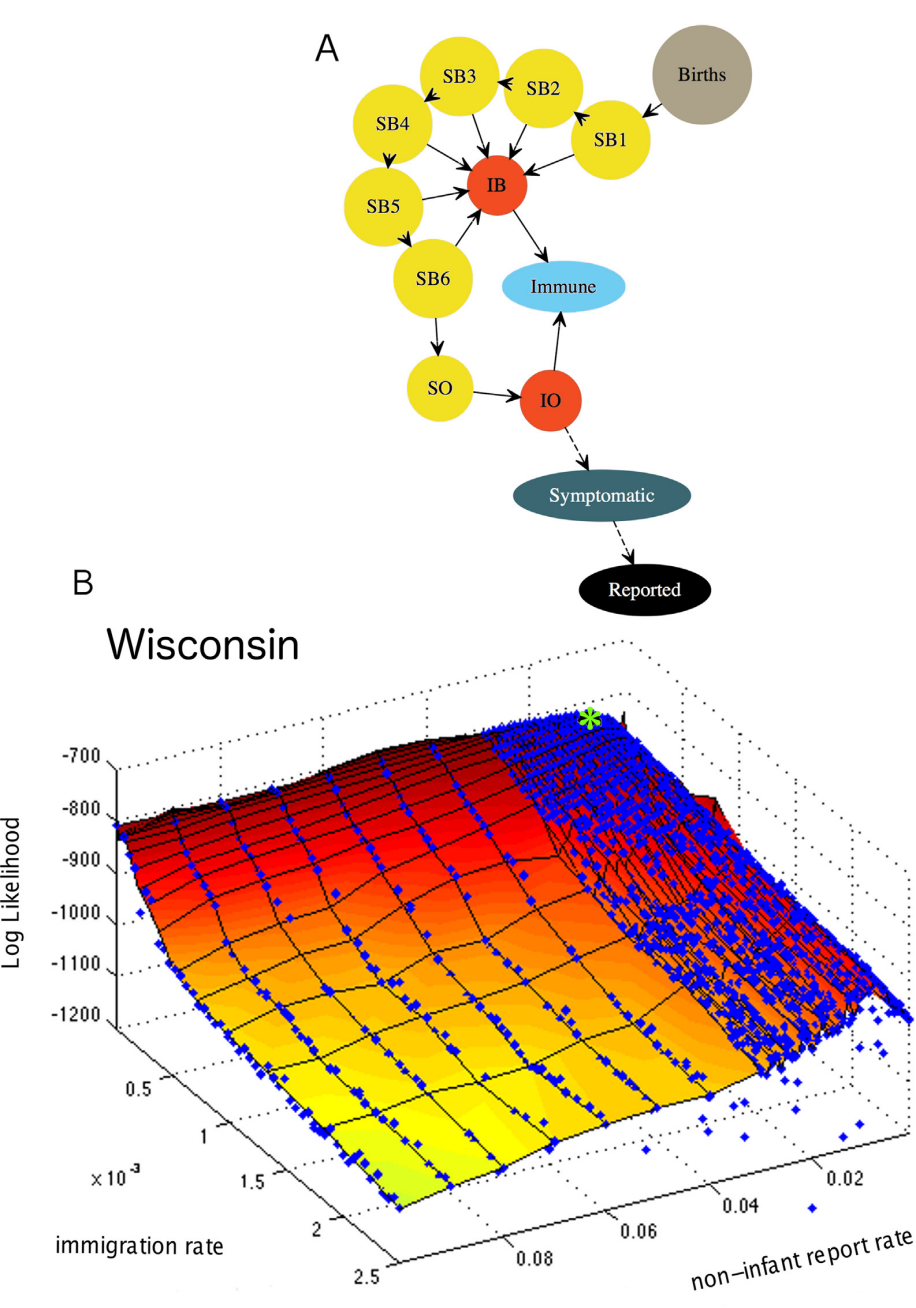
Model schematic and example likelihood profile. (A) Births enter the first susceptible infant class, $S_{1}^{B}$. Susceptible infants of age 0–6 mo, $S_{1-6}^{B}$, are susceptible to infection but are protected from symptomatic disease by maternal antibodies. Susceptible individuals over 6 months of age are in the *S* ^*O*^ class. Infected infants and noninfant infections are in *I* ^*B*^ and *I* ^*O*^, respectively. Infected individuals over 6 months of age, *I* ^*O*^, can have symptomatic illness and subsequently be reported as a clinical case with mean probability *ρ*_*t*_. *ρ*_*t*_ is a composite parameter that represents the probability of symptoms and reporting. (B) Likelihood profile for the report rate, *ρ*_*t*_, of noninfant infections and the immigration rate, *ψ*, for the state of Wisconsin. Maximum likelihood estimate (MLE) indicated by green asterisk. The report rate for Wisconsin was constant through time. The data used to make this figure can be found in S10 Data.

We constructed a dynamic stochastic model with components incorporating polio transmission, immunity, seasonality, and symptomatology along with empirical population sizes and birth rates. Birth rates displayed prominent seasonal, secular, and geographical trends (Fig S4 in S1 Text) [14]. We utilized Partially Observed Markov Process (POMP) models, which are suited for dealing with epidemiological data where the state variables (susceptible, infected, and recovered individuals) were not observed in the data; rather, the infected individuals were partially observed through clinical case reports. For our process models, we used seasonally-forced stochastic monthly discrete-time SIR models, where transitions followed a Poisson process. The infectious period was fixed at 1 mo, because multiple studies have found the duration of shedding to be 3–4 wk [15]. Infection-derived immunity was assumed to be lifelong [16,17]. The models contained six classes of infants susceptible ($S_{i}^{B}$) to infection. These infant classes contained 0–1-month-olds, 1–2-month-olds, etc., up to 6-month-olds. Models had a single infected class for infants (*I* ^B^). The older age class, which contained individuals more than 6 months of age, had its own susceptible (*S* ^*0*^) and infected class (*I* ^0^). The onset of polio symptoms ranges from 5–35 d postexposure, with a mean of 12 d [18]; therefore, we assumed reporting of symptomatic infections occurred within the 1 mo infectious period. We modeled polio reporting explicitly and, consistent with clinical evidence, assumed that maternal antibodies protected from severe disease and resulted in unreported infant infections [19–23]. Thus, we assumed that infections in individuals under 6 months of age were asymptomatic, and only individuals over 6 months of age could be symptomatic and reported as a clinical case. See model schematic in Fig 2A. The force of infection was modeled as,

$$\lambda_{t}=\left(\beta_{t}\frac{I_{t}^{O}+I_{t}^{B}}{N_{t}}+\psi \right)\varepsilon_{t}.$$ (1)

The first term of the force of infection, $\beta_{t}\frac{I_{t}^{O}+I_{t}^{B}}{N_{t}}$, represents transmission that occurred locally by individuals infected in the state at time *t*. The second term, *ψ*, encompasses WPV infection from external sources that were divorced from the local infection dynamics. *ψ* placed a lower bound on the force of infection, allowing WPV to rebound in the face of local extinction. We interpret *ψ* as indicating WPV imported from other geographic regions; however, it could also be interpreted as representing a small number of individuals in the population that shed WPV for an extended period or environmental sources helping WPV persist over the winter. The transmission parameter, *β*_*t*_, was parameterized using a B-spline, providing it the flexibility to have a constant or seasonal transmission rate. There was seasonality, but no interannual variation, in the transmission rate,

$$\beta_{t}=\text{exp}\sum_{i=1}^{6}q_{i}\xi_{i_{t}}.$$ (2)

Here, each $\xi_{i_{t}}$ is a periodic B-spline basis with a 1 y period. The process noise, *ε*_*t*_, was gamma distributed with mean 1 and variance that scaled to account for both environmental and demographic stochasticity; refer to S1 Text, Equation S6 for further details. We assumed cases were drawn from a rounded, left-censored normal distribution with a mean report rate of *ρ*_*t*_ and dispersion parameter *τ*,

$$\begin{array}{c} {\text{cases}}_{t}=\text{round}(x_{t}),\;x_{t}\sim \text{normal}(\rho_{t}I_{t}^{O},\tau I_{t}^{O}). \end{array}$$ (3)

For calculating the likelihood, we used a binned-normal probability density. Full model details are found in S1 Text, Section S1.3.

We fitted SIR models (one for each state in the US) to data independently using Maximization by Iterated particle Filtering (MIF) in the R package pomp [24–26]. For each state, we estimated 14–15 parameters. The parameters estimated were: 6 seasonal transmission parameters (β_*i*_), 3 parameters accounting for process and measurement noise, 3 initial conditions for the older age class, the external contribution to the force of infection (*ψ*), and 1–2 report rates (*ρ*_*t*_). MIF is a simulation-based likelihood method for parameter estimation. The basis of MIF is particle filtering, which integrates state variables of a stochastic system and estimates the likelihood for fixed parameters. Instead of fixing parameters, MIF varies them throughout the filtering process and selectively propagates particles (i.e., parameter sets) that have the highest likelihoods. By initializing MIF at a variety of points distributed across parameter space, we estimated the shape of the likelihood surface for each US state and identified the Maximum Likelihood parameter Estimates (MLEs). MIF was initialized from 1 million parameter sets for a global search, followed by additional phases of increasingly localized searches, which included profiling. In total, for each US state, MIF was initialized from more than 10,000 locations in parameter space to estimate the shape of the likelihood surface and identify the MLEs.

Prior to 1945, nonparalytic polio cases were rarely included in our data, but the reporting of nonparalytic polio became increasingly common [1,3]. Thus, we tested an optional parameter to account for increased representation of nonparalytic polio in clinical cases data. We estimated two report rates, one for the pre-baby boom era and another for the baby boom era, and discriminated between models with and without time-varying reporting using Akaike Information Criterion (AIC). Profiles were constructed for the two versions of the model, one in which the report rate was constant through the entire time period and one in which the report rate increased during the baby boom era. For each state, AIC was used to discriminate between constant and time-varying reporting, and the MLEs were drawn from the appropriate two-dimensional profile. Inference was performed using the data from May 1932 to January 1953, with the exception of South Dakota and Texas, for which the first data used were from May 1934, and May 1935, respectively, i.e., May of the year following the first full year of available data, the lag being needed to estimate initial conditions for the infant classes directly from birth data. For model validation, the last two epidemic years were set aside for forecasting. Full details are provided in S1 Text, Section S1.4. Likelihood profiles were constructed for each US state (example in Fig 2B, all others in Fig S9–S17 in S1 Text).

**Fig 3:**
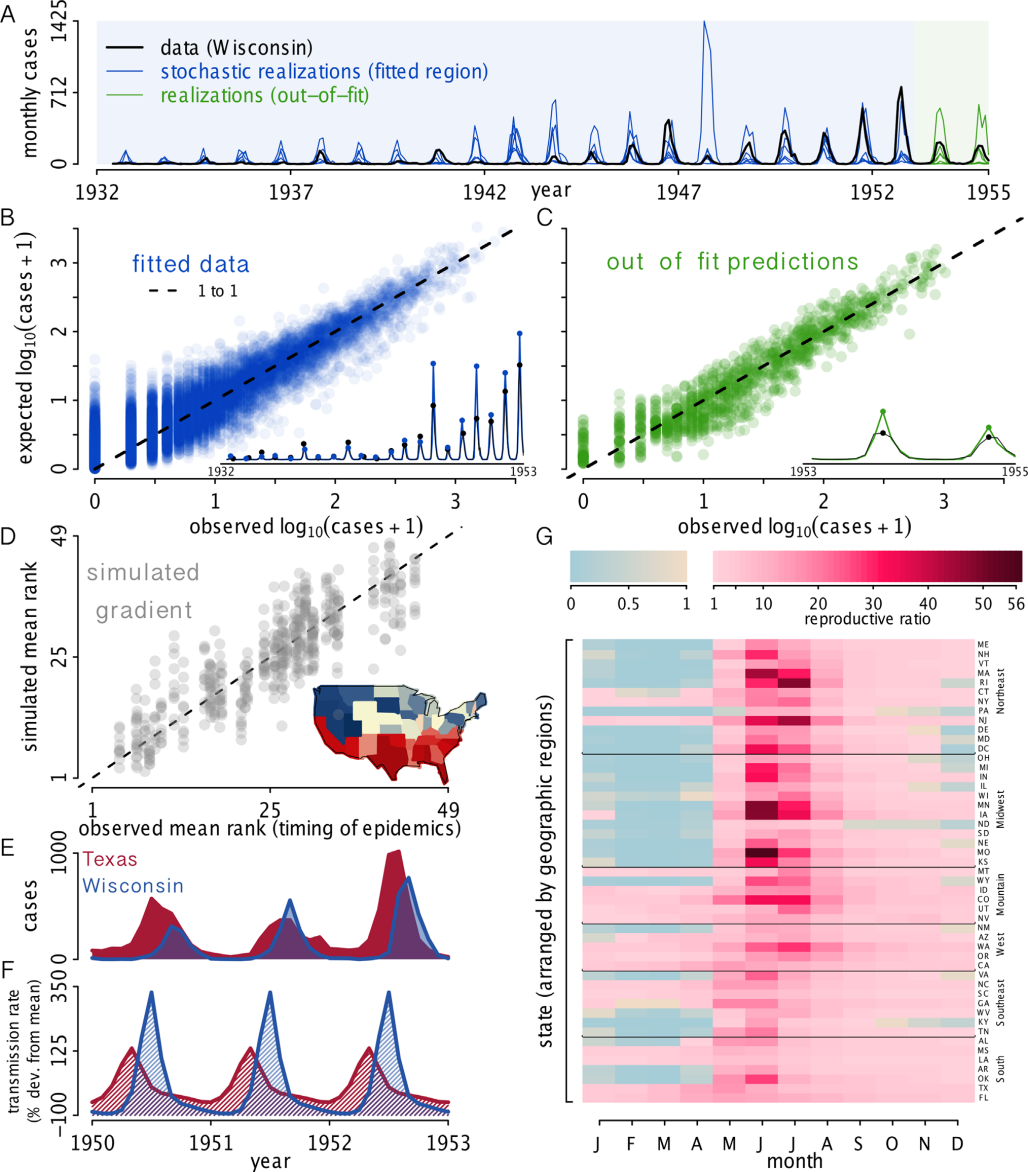
Fitted model and seasonality. (A) Observed data (black, shown for Wisconsin) and three stochastic simulations from the MLE (blue and green) highlight that both observed and simulated polio epidemics have a large amount of interannual variation in size but a narrow seasonal window. Fitted and out-of-fit data regions are indicated by blue and green, respectively. (B) Model validation showing observed log_10_(cases) versus expected log_10_(cases) for fitted data and (C) out-of-fit predictions for all 49 states. Expected cases are one-step-ahead predictions from the fitted models. Insets show observed cases (black) and expected cases (blue and green) for Wisconsin. Fitted data include May 1932–January 1953 for all states except South Dakota and Texas, whose covariate data limited our inference to begin in May 1933 and 1934, respectively; out-of-fit data spanned January 1953–December 1954. The generalized *R* ^2^ = 0.76 for the fitted data and *R* ^2^ = 0.61 for out-of-fit data, calculated on the natural scale, while data are plotted on a log scale for visualization. (D) Observed versus simulated mean rank of epidemic timing based on ten realizations of the fitted models. Inset shows the latitudinal gradient from one simulation; colors match Fig 1E and 1F. (E) Monthly polio cases in Texas and Wisconsin and (F) the MLE transmission rates. Epidemics occured earlier in southern states than northern states because the seasonal peak in transmission occured earlier at lower latitudes. (G) MLEs of the seasonal transmission rate for each state organized by geographic region; in our models, this represents the reproductive ratio. The reproductive ratio varies both seasonally and geographically, with some states having a reproductive ratio less than 1 during the wintertime off-season. The data used to make this figure can be found in S1 Data, S4 Data, S8 Data, S11 Data, and S12 Data.

**Fig 4:**
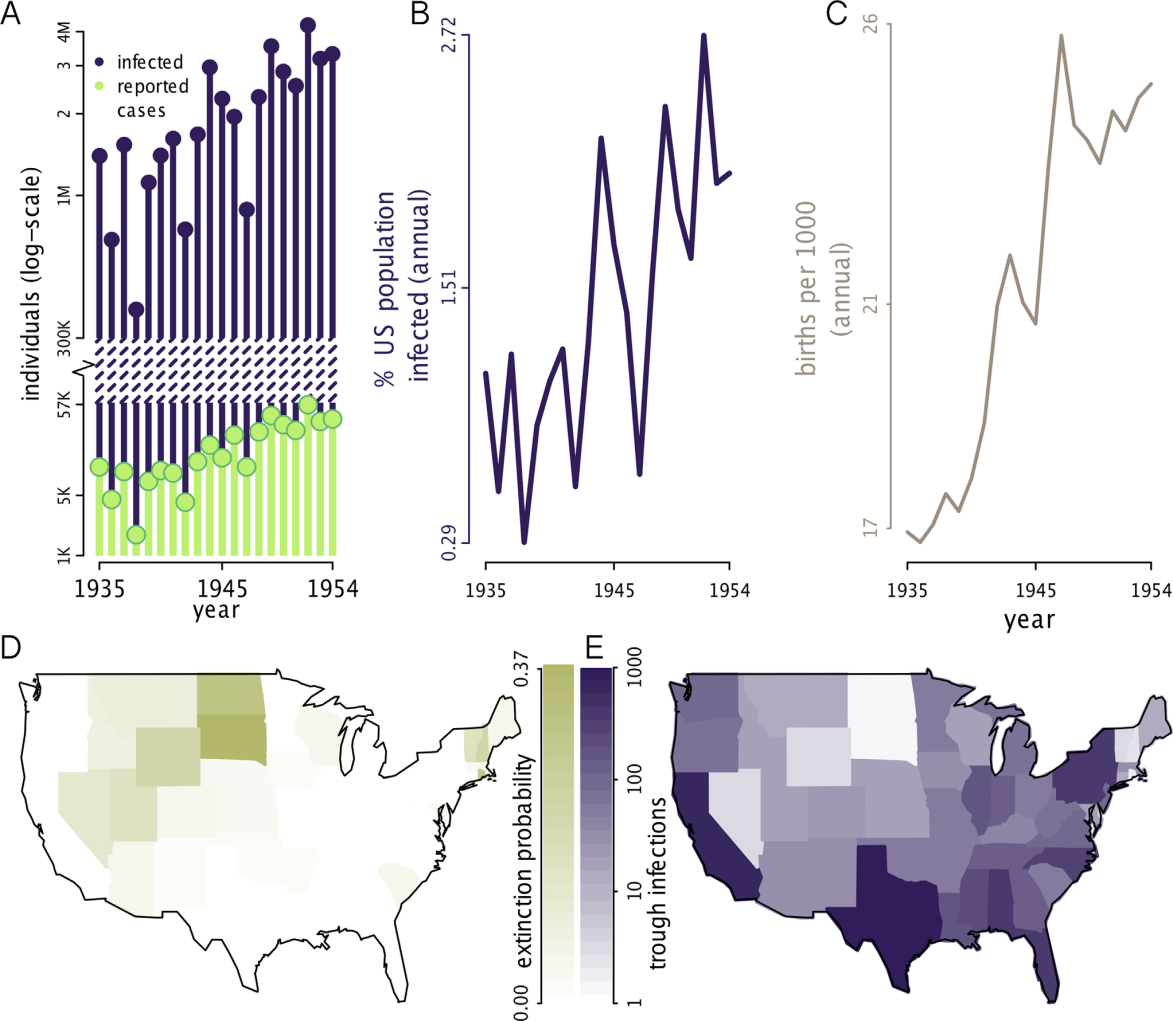
Epidemic emergence and source-sink dynamics. (A) Annual number of infected individuals in contrast to the small number of reported cases. Annual infections were reconstructed for the US using particle filtering means. The particle filtering mean is the expected value at time *t*, given the data up to time *t*. (B) Annual infections in the US represented as the percent of the population. Reconstructed infections show an increase in infection incidence that accompanies (C) the increase in the birth rate. (D) Simulated WPV extinction probability. The probability of extinction measured as the mean annual probability of observing an extinction during the off-season (December–May). “Sink" populations are those states with a high extinction probability. (E) Simulated trough infections. Trough infections indicate the minimum number of infections during off-seasons. For each US state, the median was taken across simulations and averaged across years. “Source" populations are those that maintain a high number of infections. Panels D–E were constructed using the 500 stochastic simulations for each state. The data used to make this figure can be found in S1 Data, S4 Data, S5 Data, S6 Data, S7 Data, and S9 Data.

**Fig 5:**
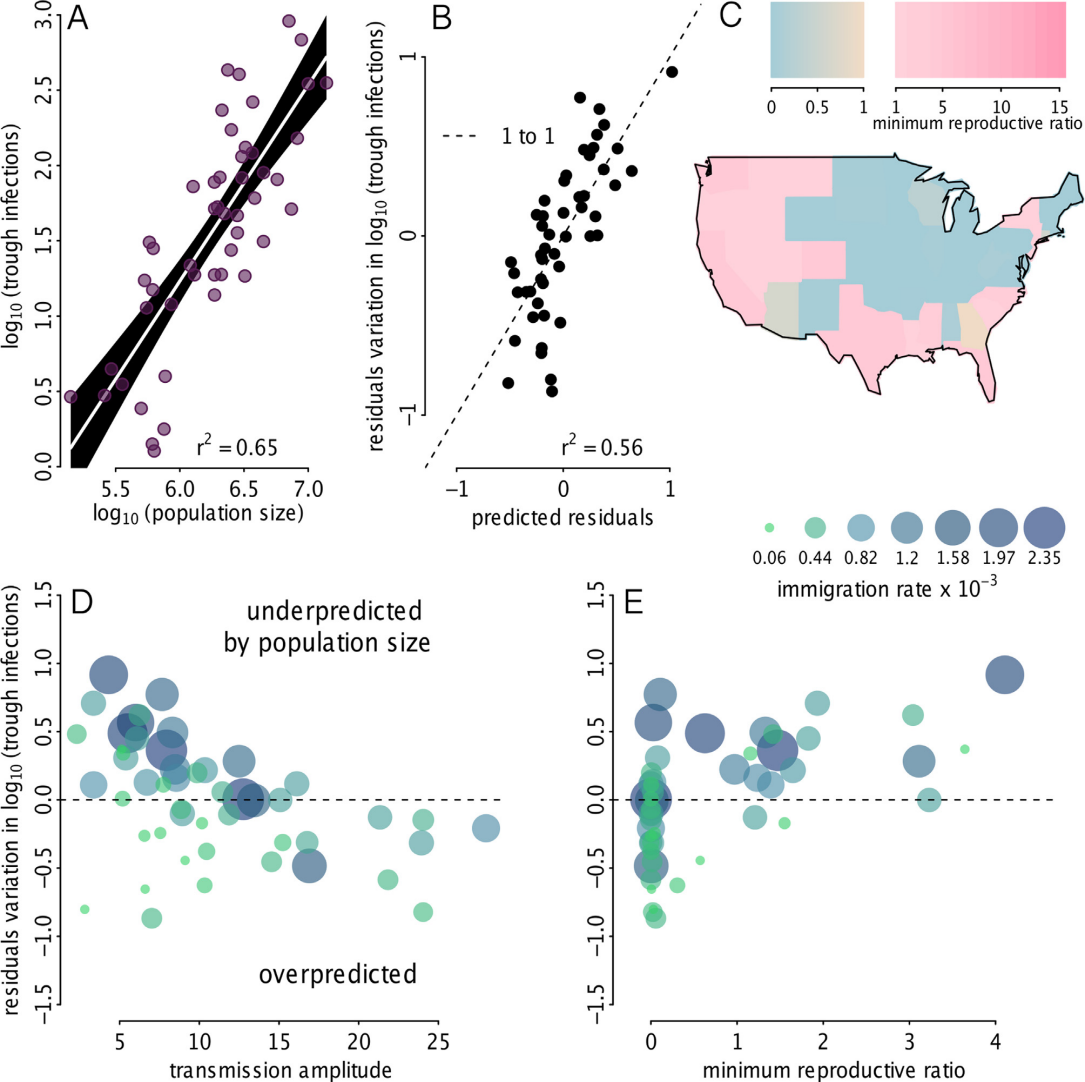
Source-sink population predictors. (A) Linear regression of state population size versus simulated trough infections, both on a log_10_ scale. Trough infections are those shown in Fig 4E. (B) Residuals from the regression of population size versus trough infections were used as the dependent variable in the multiple regression model, where the predictors were: the state’s seasonal minimum reproductive ratio, the immigration rate, and the seasonal amplitude of the reproductive ratio, measured as half the peak–trough difference in the reproductive ratio. Plot shows on the *y*-axis, the residuals, *r*_*i*_, from panel A, along with the prediction of the residuals based on the multiple regression $r_{i}=b_{0}+b_{1}\text{min}(R_{t}^{i})+b_{2}\frac{\text{max}(R_{t}^{i})-\text{min}(R_{t}^{i})}{2}+b_{3}\psi_{i}$, where $R_{t}^{i}$ is the reproductive ratio, *ψ*_*i*_ is the immigration rate, and *i* indicates the state. Taken together, panels A and B demonstrate that the predictors of a source versus sink are: the population size, the minimum reproductive ratio, the amplitude of the reproductive ratio, and the immigration rate. (C) Map of the seasonal minimum reproductive ratio showing geographic clustering. (D) The residuals, *r*_*i*_, versus the seasonal amplitude of the reproductive ratio (i.e., the transmission amplitude), point size and color indicate the immigration rate, *ψ*_*i*_. (E) The residuals, *r*_*i*_, versus the seasonal minimum reproductive ratio, point size and color indicate the immigration rate, *ψ*_*i*_. The data used to make this figure can be found in S3 Data, S4 Data, S5 Data, S6 Data, S7 Data, and S8 Data.

**Fig 6:**
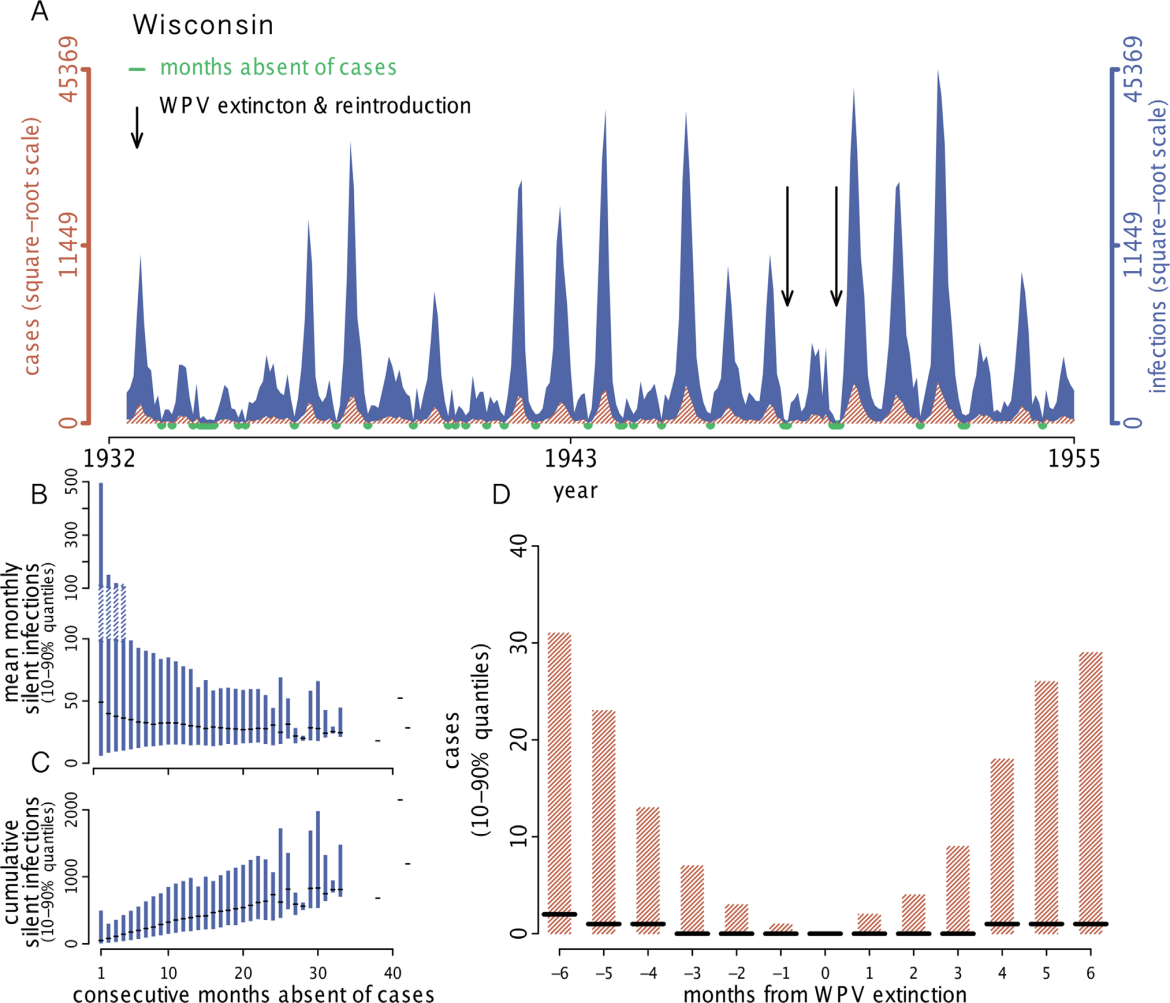
Persistence mechanisms. (A) Example of simulated infections and cases for Wisconsin. Months absent of reported cases are indicated in green. During periods when the disease is absent, WPV infections are often silently transmitted in the population. In this simulation, there were two instances (indicated by arrows) when the local chain of transmission was broken and WPV went locally extinct but quickly rebounded due to reintroduction. This example illustrates the two polio persistence mechanisms observed throughout the US, which are (i) local WPV persistence via unbroken chains of transmission and (ii) WPV extinction and reintroduction. (B) Distributions of mean monthly silent infections during periods absent of reported disease. (C) Distributions of cumulative silent infections during periods absent of disease. Distributions in B and C are 10%–90% quantiles and the median, based on 500 simulations per state. Silent infections are those that occur in the absence of reported cases and highlight the unobservable dynamics of polio. (D) Simulated cases surrounding WPV extinction events. Distributions show 10%–90% quantiles and the median number of cases observed up to 6 mo preceding and 6 mo following an extinction event. Generally, fewer than 5 cases/mo are reported 2 mo to either side of an extinction event. However, it is unclear whether 5 mo, each with less than 5 cases, is a reliable signal of extinction. The data used to make this figure can be found in S4 Data, S5 Data, S6 Data, S7 Data, S13 Data, and S14 Data.

To quantify model–data agreement, we evaluated the accuracy of one-step-ahead predictions for all 49 states, both for data used in model parameterization (Fig 3B) and for out-of-fit data (Fig 3C). Because of correlations between states (which vary significantly in size and mean incidence), simple linear regression is not appropriate for assessing model–data agreement; therefore, generalized *R* ^2^ was calculated to quantify the proportion of the variance explained by the model relative to that explained by state alone. We calculated the generalized *R* ^2^ for the one-step-ahead predictions and out-of-fit predictions (See S1 Text, Section S2.2 for details). For Fig 4A and 4B, infections were reconstructed using particle filtering means, and the reconstruction was limited to data beginning in Jan 1935, because 1935 is the first full year for which we have the models parameterized for all states. Following model validation and infection reconstruction, the fitted models were used as simulation tools to explore polio infection dynamics. In Fig 4D and 4E, we used 500 simulations for each state from 1935 through 1954. In Fig 4D, we present the state-specific probability of extinction by examining 500 realizations of the fitted models. Specifically, we calculated the annual probability of polio extirpation during the off-season (December–May) and averaged across years. Similarly, in Fig 4E the minimum number of infections during each off-season was based on 500 simulations. For each simulation, the annual minimum number of infections was identified, and the median was taken across the 500 simulations and averaged across years. In order to identify the covariates and epidemiological parameters that influenced the number of trough infections—a measure of WPV persistence—we regressed trough infections with various covariates and parameters; results shown in Fig 5. In Fig 6B–6D, distributions were generated by characterizing observations across 500 simulations per state. All simulations and data used for producing the figures in this manuscript are available in S1 Data–S14 Data.

## Results

### Polio’s Seasonality and Latitudinal Gradient

In the mid-20th century, polio outbreaks in the US were strongly seasonal. Epidemic peaks typically occurred between August–October (Fig S1 in S1 Text); but the magnitude was highly variable among states. In the transition from the pre-baby boom era (1931–1945) to the baby boom (1946–1954), epidemics increased in size and became more regular (Fig 1A and 1B). Winter troughs were frequently marked by consecutive months without reported cases. During the baby boom, the frequency of these local fadeouts diminished (Fig 1C), while epidemics became more tightly synchronized (Fig 1D). There was a striking latitudinal gradient in the timing of epidemics across the entire country (Fig 1E and 1F, Fig S1 in S1 Text). Two broad classes of mechanisms can give rise to such a pattern. Seasonal movement of the pathogen from southern populations can generate a traveling wave, which has previously been observed in measles [27], dengue [28], influenza [29,30], and pertussis [31]. Alternatively, the pattern may indicate latitudinal gradients in demographics (e.g., birth rates [14,32]) and/or environmental factors associated with transmission.

### Model Fit

Our extensive search of parameter space resulted in the MLEs for each parameter. To quantify the shape of likelihood surface along two parameter dimensions we identified as important (i.e., the report rate, *ρ*_*t*_, and the external contribution to the force of infection, *ψ*), we constructed two-dimensional likelihood profiles for each US state. Two-dimensional profiles, by definition, have fixed parameter values along two dimensions of parameter space, while the likelihood is maximized along all other parameter dimensions. There were 12 states that had constant reporting (i.e., the same report rate during the pre-baby boom and baby boom era). Fig 3A illustrates that the fitted models generate epidemic trajectories that display: (1) the seasonal characteristics of polio and (2) the large amount of interannual variation in epidemic size. Importantly, the fitted models faithfully reproduce observed dynamics. In particular, the seasonality, epidemic shape, interannual variability in epidemic magnitude, and the increase in incidence during the baby boom are captured by the models. Model fit was formally validated using one-step-ahead predictions for all 49 states (Fig 3B) and out-of-fit predictions (Fig 3C), which indicate good agreement between models and data. Furthermore, geographical structure in the timing of observed epidemics is captured by the fitted models (Fig 3D). State-specific examples of one-step-ahead predictions and out-of-fit predictions are shown in Fig S2 and S3 in S1 Text.

### Explaining the Latitudinal Gradient

We hypothesized that the latitudinal gradient in epidemic timing was driven by either: (1) geographic variation in transmission because of environmental factors that modulated transmission, (2) the geographic trend in birth seasonality in the US (detailed in [14]), or (3) the movement of pathogen from south to north.

In support of hypothesis 1 (i.e., environmental factors), we identified a spatial pattern in the phase of seasonal transmission (Fig 3E–3G, Fig S7 in S1 Text). States with earlier epidemics had an earlier peak in the seasonal transmission rate in the fitted models. Interestingly, because of polio’s long infectious period, peaks in transmission preceded incidence peaks by 1–2 mo. States varied geographically not only in the timing of the transmission peak but also in the wintertime transmission trough depth and trough duration (Fig 3G).

Epidemiological theory indicates that birth seasonality can have important dynamical consequences for childhood diseases [14,33,34]. To test hypothesis 2 (i.e., birth seasonality), we carried out a comparison of the fitted models with and without birth seasonality. Simulations of both models expressed the latitudinal gradient (Fig S5 in S1 Text). Therefore, birth seasonality is not necessary to explain the polio gradient because geographic variation in transmission is sufficient. We attribute the negligible effect of birth seasonality on polio incidence to the low amplitude of birth seasonality, which was approximately 10% in the US at this time.

We suggest that hypothesis 3 (i.e., pathogen movement) is an unlikely explanation of the latitudinal gradient. If the latitudinal gradient were a wave of pathogen movement, it would require a high wave speed, which we see as incompatible with transport of the pathogen across the landscape. The pattern in Fig 1E and 1F corresponds to a wave traveling approximately 1,200 km/mo. For comparison, waves in pertussis have been estimated to travel 110–320 km/mo [31]; waves in dengue appear to move 150 km/mo [28]; and the measles wave speed in the United Kingdom was estimated at 20 km/mo [27]. A polio wave that is 10-fold faster than pertussis in the US is difficult to justify and unnecessary, because our fitted models support hypothesis 1. Thus, we have determined that polio’s latitudinal gradient is driven by geographic variation in transmission; we are left with an unidentified seasonal driver that modulates transmission.

While geographical variation in birth seasonality was insufficient to explain the latitudinal gradient seen in epidemic timing, birth seasonality had a small but observable effect on the simulated incidence of infant infections. To quantify the influence of birth seasonality on infant infections, we compared simulations of the fitted models to simulations for which seasonal fluctuations in births were removed. In the presence of birth seasonality, infant infection incidence was often higher (Fig S6 in S1 Text); however, this did not affect the incidence of disease directly, and no indirect effect was observed.

### Symptomatology

It is well known that AFP incidence represents a small fraction of true WPV prevalence [35,36]. Reassuringly, our independent estimates from the incidence data agree: our MLEs indicate that typically less than 1% of polio infections were reported. We assumed that infected infants under 6 months of age were asymptomatic, due to protection by polio-specific maternal antibodies. The report rate for individuals not maternally-protected was 0.75% (averaged across states) in the pre-baby boom era and rose to 1.4% in the baby boom era, with considerable variation across states (Fig S8 in S1 Text). Overall, we estimate that there were often over 1 million annual infections in the US; though only 2,000–57,000 cases were reported every year (Fig 4A). Our results are in line with a 1948 serology-based study in North Carolina, which estimated 62–175 subclinical polio infections per paralytic case [37].

### Spatiotemporal Heterogeneity in the Reproductive Ratio

The fitted models revealed vast seasonal and spatial heterogeneity in WPV’s reproductive ratio. Fig 3G shows large seasonal fluctuations in the reproductive ratio within each state. Several states maintained a reproductive ratio above 1 throughout the year. In contrast, 28 states had reproductive ratios that fell below 1 for 4–5 mo from December–April.

States in the Northeast and Midwest had extreme seasonal variation in their reproductive ratio. Deep winter troughs in transmission in the Northeast and Midwest often had several consecutive months with a reproductive ratio below 1. In contrast, at the peak of transmission in June and July, these same states had a reproductive ratio above 20. Interestingly, each geographic region other than the Midwest had at least one state that maintained a reproductive number above 1 throughout the year. Southern states typically maintained an intermediate transmission rate throughout the year.

### Epidemic Emergence

Our analyses provide a new perspective on polio’s historical emergence. Commonly described as a “disease of development,” polio’s emergence has been ascribed to improved hygiene that reduced transmission and pushed the burden of infection onto children more susceptible to paralytic polio. This explanation requires that reduced transmission raised the mean age of infection and therefore the risk of AFP [1]. Our results suggest the marked increase in polio incidence from the 1930s to the 1950s was a straightforward consequence of increased birth rates (Fig 4B and 4C), and that hygiene effects on transmission are not required to explain polio’s rise to epidemic levels. Since polio’s epidemic emergence was captured in the models as a consequence of the changing birth rate, we did not explicitly test reductions in the transmission rate as an additional contributor to epidemic size, and we cannot completely rule out trends in transmission as a contributing factor. While the “disease of development” explanation has also been questioned on other grounds [22], changes in hygiene and sanitation could have contributed to the initial emergence of polio, which occurred from the late 1800s to the early 20th century.

### WPV Persistence

Polio cases were consistently observed throughout the US during the period of this study. We hypothesized: (a) WPV persisted locally in each state, or alternatively, (b) WPV regularly went locally extinct and reinvaded from elsewhere. Due to polio’s high asymptomatic infection ratio, distinguishing between these two mechanisms of persistence cannot be done using reported cases alone, since WPV may be present during the off-season even in the absence of clinical cases. In order to determine which of these two persistence mechanisms was the likely explanation of continued infection, we simulated the fitted models and characterized the dynamics of the process models (i.e., the unobserved infection dynamics rather than the observable disease dynamics). We focused on determining whether infections persisted during the wintertime off-season or if extinction and reinvasion occurred. In particular, we assessed (i) the average annual probability of an extinction event in each state, which results from diminished local transmission and (ii) the annual minimum number of infections. Fig 4D and 4E depict the geographic variation in these quantities. Some states experienced frequent local extinction during the off-season, followed by recolonization; we consider these “sink” populations. In contrast to sink states, a few states maintained infections year-round; these we define as “source” populations. The majority of states, however, were neither consistently sources nor sinks, because even sink states had frequent overwintering of WPV. The fitted models suggest that WPV underwent extinction and recolonization in the classic metapopulation sense.

### Source-Sink Population Predictors

We explored characteristics that contributed to states having been WPV sources versus sinks. We used simulated trough infections, shown in Fig 4E, as the indicator of a source versus a sink. States that maintained a high number of trough infections enabled WPV to persist through the off-season; whereas states with a low number of trough infections were likely to have experienced regular WPV extinction. State population size accounted for 65% of the variation in the number of trough infections (Fig 5A). We used multiple regression models to determine whether the (i) mean birth rate, (ii) amplitude of birth seasonality, (iii) immigration rate, (iv) seasonal minimum reproductive ratio, and/or (v) seasonal amplitude of the reproductive ratio explained the residual variation in trough infections, after controlling for population size. The mean birth rate and amplitude of birth seasonality had a negligible impact on the residual variation in trough infections; therefore, they were removed from the multiple regression model. A multiple regression model with the immigration rate, seasonal minimum reproductive ratio, and the seasonal amplitude of the reproductive ratio explained 56% of the residual variation in trough infections (Fig 5B). Interestingly, even though there were no clear geographic patterns of source-versus-sink localization (Fig 4D and 4E), there was strong geographic clustering in the minimum reproductive ratio (Fig 5C), demonstrating that even though source-sink predictors display geographic clustering, the combination of predictors can generate a source-sink mosaic. We found that after accounting for population size, states with a higher immigration rate had more trough infections (Fig 5D and 5E). States with a higher transmission amplitude, however, had fewer trough infections; we interpret this as being due to susceptible depletion followed by deep infection troughs in states with a high transmission amplitude (Fig 5D). The minimum reproductive ratio had a positive relationship with trough infections; states that maintained a reproductive ratio above 1 during the off-season tended to have more trough infections during the off-season (Fig 5E).

### Silent Infections

Disease eradication programs face the significant challenge of verifying success in the presence of asymptomatic infections. Typically, a criterion for success is the absence of disease for an extended period; however, the utility of this criterion is questioned when the symptomatic cases reported are only the tip of the iceberg in terms of infection. Using our fitted models, we explored the reliability of absence-of-disease as an indicator of WPV extinction. Because of widespread subclinical infections, there was a stark contrast between the simulated number of polio infections and clinical cases (Fig 4A). This contrast (i.e., the disconnect between infections and clinical cases), can lead to epidemiological scenarios where absence-of-disease is uninformative. In our models, WPV persistence was achieved by one of two mechanisms: (1) local unbroken chains of transmission, or (2) local extinction followed by rapid reintroduction. For each of these two mechanisms, we found that clinical case data can be misleading, as outlined in Table 1. For instance, if WPV circulated at low levels of infection, extended absence of clinical cases could lead to the conclusion that WPV was locally eradicated. Similarly, if local extinction of WPV occurred, and was quickly followed by reintroduction and clinical cases, local extinction could go unrecognized, potentially misdirecting targets for control (e.g., to focus on sink populations rather than source populations).

**Table 1:** Four scenarios for the relationship between WPV infections and clinical disease.

	Local Persistence of WPV	Local Extinction and Reintroduction
Extended absence of disease	Disease data are uninformative, and potentially misleading, because WPV is circulating silently via subclinical infections	Disease data reflect that WPV goes extinct and is reintroduced
Disease observed regularly	Disease data reflect that WPV persists and transmission is ongoing	Disease data are uninformative because they mask that WPV goes extinct and is reintroduced

Local persistence of polio—within a state, region, or country—occurs when WPV overwinters during the off-season and the transmission chain is unbroken year-round. In contrast, local extinction and reintroduction occurs when WPV goes extinct during the off-season, breaking the chain of transmission; a new transmission chain begins when WPV is reintroduced from elsewhere. Discriminating among these scenarios is necessary for planning eradication strategies in endemic regions.

By simulating our fitted models, we identified extended periods absent of disease and used these periods to quantify the number of silent infections (Fig 6B and 6C). We observed that if infections were maintained at relatively low numbers (i.e., under 100 infections per month), then WPV could circulate silently for over 30 months (Fig 6B). The silent circulation of WPV can result in thousands of infections before a single reported case is observed (Fig 6C). Our models assumed homogeneous mixing within each US state, and it is important to recognize that different mixing patterns could increase or decrease the lengths of chains of silent transmission. Because of the silent circulation of polio, it is difficult—and perhaps indefensible—to use clinical case data (i.e., without fitted models) to evaluate WPV persistence. We simulated the fitted models to quantify the distribution of cases observed during periods with WPV extinction (Fig 6D). The distribution of cases surrounding WPV extinctions is fairly symmetric because of the reintroduction of WPV following extinction. Therefore, we conclude that, in the face of rapid reintroduction following WPV extinction, case data cannot be used to identify extinction events. Though it is desirable to use fitted models to identify signals of extinction, and apply this knowledge to case data, it would require extensive evaluation of silent circulation.

## Discussion

This work sheds light on the fundamental ecology of WPV. Latitudinal gradients have been identified in several acute viral infections, including influenza, Respiratory Syncytial Virus (RSV), rotavirus, and now polio [38,39]. Our results indicate that the observed latitudinal gradient in the timing of polio epidemics is driven by a latitudinal gradient in demographic and/or environmental factors associated with transmission. Determining which mechanism is responsible has implications for control and surveillance efforts. Specifically, knowledge of the seasonal driver could allow for regionally-timed national immunization campaigns or the ability to forecast changes in epidemic seasonality.

Our identification of birth rate as a driver of polio’s epidemic emergence during the baby boom of the 1940s and 1950s is yet another demonstration [40,41] of the need for full integration of demography into the study of childhood infectious disease epidemiology. The rate of susceptible recruitment has long been known to control the magnitude and frequency of epidemics of fully immunizing childhood diseases [40,42]. Today, in an era of human population expansion and emerging infectious diseases, we are reminded of the importance of characterizing changes in host population ecology. As a result of limits of our demographic data, we were unable to address the early emergence phase of polio from the late 1800s through the 1920s. Rather, we focused on the later phase of emergence in the US, as the disease transitioned from small epidemics in the 1930s and early 1940s to large epidemics during the baby boom era. Though there were increases in the report rate, which contributed to the trend in observed cases, we also discovered an increase in the incidence of infection. Importantly, the increase in infection incidence closely tracked birth rates in the mid-1900s.

To the extent that our results bear on contemporary polio ecology, the identification of source-sink dynamics in the US suggests that successful local elimination of polio in a sink population is inconsequential in the presence of a source population. This prediction has unfortunately been repeatedly borne out in current epidemics. Regional elimination of polio has been followed by reintroduction from endemic countries, such as the 2013 outbreak in Somalia, Ethiopia, and Kenya, with WPV introduced from Nigeria and repeat reinfection of Afghanistan from Pakistan [43]. Moreover, the metapopulation structure of WPV demonstrates that preventing emigration of WPV from source populations—which may be highly localized—is a requirement for efficient control.

We estimate that over 99% of infections were subclinical, with the reporting of total infections regularly below 1%. Importantly, subclinical infections are likely more common today than in the period we studied. This is because, first, both nonparalytic and AFP cases were reported in the US, whilst only AFP cases are currently reported. Second, our models were fit to data during the vaccine-free period of polio endemicity; therefore, infection incidence was elevated each summer, allowing the number of infections to grow sufficiently large to result in a high probability of clinical infections. In contrast, today, as polio’s reproductive number approaches *R*_t_ = 1 in highly vaccinated endemic countries, WPV can circulate at levels below the level needed for likely clinical observation. The recovery of environmental WPV isolates in Israel in the complete absence of AFP cases supports this expectation [43]. Furthermore, Fig 6B demonstrates that polio may circulate silently for extended periods (i.e., longer than 3 y) if the number of infections remains below the threshold for likely detection. Two years of silent WPV circulation has been confirmed: The outbreak in Central Africa detected in October 2013 was traced back to WPV circulation in Chad during 2011 [44]. Populations expected to have a small number of monthly infections in the presence of WPV—because of their demography or because they are highly vaccinated—would therefore be desirable targets for intense environmental surveillance. In terms of information gained, environmental surveillance is a powerful tool for identifying silent transmission in locations where polio would otherwise go undetected. In Pakistan, the level of environmental surveillance has increased since 2011, and WPV has consistently been detected, even in the absence of AFP cases [45].

In the absence of validated transmission models, case data are relied upon to determine whether a pathogen has gone locally extinct and estimate the critical community size required for pathogen persistence. In light of polio’s propensity for silent circulation, we conclude that AFP data can be misleading; this conclusion extends to any communicable disease in which clinical cases represent a small fraction of infections. Extended periods absent of reported cases can mask infections circulating at levels below the threshold for likely reporting. We therefore advocate fitting transmission models to contemporary data to draw inferences regarding extinction. Since infection can persist even in the extended absence of reported cases, knowledge of the local infection dynamics could reveal invaluable epidemiological information. Transmission models fit to endemic countries (i.e., Pakistan, Afghanistan, and Nigeria) could be used to identify how demographic and environmental factors interact with vaccine coverage to determine regional WPV persistence. In addition to coupling case data with transmission models for endemic countries, another useful extension would be to combine genetic data from WPV isolates with transmission models to further distinguish between sustained local transmission and imported infection. Genetic studies have found reductions in WPV genetic diversity in Afghanistan, suggesting local extinction of some WPV strains [6].

Vaccination campaigns might take further advantage of the seasonality and geographic clustering of WPV’s reproductive ratio. Low-transmission-season vaccination campaigns have been utilized by the Global Polio Eradication Initiative (GPEI) [6]. We found that the “low season” reproductive ratio can have geographic clusters where the reproductive ratio is greater than 1, which, if identified in the contemporary setting, might be useful targets for intense low season vaccination campaigns. Additionally, if the: (1) seasonal reproductive ratio, (2) birth seasonality, and (3) vaccine coverage are quantified for endemic countries, vaccination campaigns could use this information to determine the regionally optimal timing for national vaccination days. These three quantities could be used to estimate the seasonal effective reproductive number and evaluate alternative vaccination strategies. For instance, one strategy might be to extend the duration of the wintertime trough (i.e., by generating or extending the window during which the effective reproductive number is below 1), which may push WPV to extinction. Alternative strategies might be to vaccinate in the months prior to the seasonal peak in transmission or six months following the peak in births. In the past, mass OPV campaigns held during the low transmission season were deemed “most effective" [46], but it is unclear to what extent this strategy is used today.

Historical data, particularly in pre-vaccine periods, offer a unique glimpse into the ecology of infection, without a high degree of human intervention. Historical data offer several advantages. First, reporting rates from historical eras are informative because they are reflective of (a) the symptomatology of infection and (b) clinical diagnosis of symptomatic infection. Second, it can be difficult to infer unobserved infection dynamics using data for diseases that are near their eradication or elimination threshold. This is because the parameterization of transmission models with data containing few cases—and lacking recurrent epidemics—can result in ambiguous parameter estimates. The recurrent nature of historical epidemics gives us the unique opportunity to unravel disease-specific transmission ecology. Once the baseline transmission ecology is known, it can be coupled with data from contemporary periods to test hypotheses regarding modern day epidemics and their geographic coupling.

Our analyses demonstrate the power of an approach focused on coupling mechanistic transmission models with long-term, spatially replicated longitudinal incidence data. Specifically, we document intriguing continental-scale gradients in polio seasonality, which we suggest are explained by latitudinal gradients in local transmission rates. We also show that the historical emergence of epidemic polio was largely a consequence of demographic trends rather than improvements in hygiene. Importantly, we demonstrate that historical polio persistence in the US was driven by an ever-changing mosaic of source-sink populations. Finally, we found that even protracted AFP-free periods do not reliably indicate WPV extinction. Because of the difficulty in establishing fundamental aspects of WPV transmission in heavily vaccinated populations, it is our hope that these insights will act as a baseline for understanding modern polio transmission and disentangling vaccine effects from the natural ecology of the disease.

## Supporting Information

S1 Text Supporting information. File containing model details, extended inference methods, and results. S1 Text includes multiple figures referenced in the main text.
(PDF)

S1 Data Polio data. File containing monthly polio cases per state in the US from January 1931–December 1954, digitized from the US Morbidity and Mortality Weekly Reports. Provided by the CDC.
(CSV)

S2 Data Coordinates. File containing latitude and longitude for the state population centers based off of the year 2000 US census.
(CSV)

S3 Data Population data. File containing annual estimates of the population size for each state in the US from 1910–2008; these are the intercensal estimates by state from the US Census Bureau.
(CSV)

S4 Data Simulated infections. File containing 125 stochastic realizations of monthly infections (including both infant and noninfant infections) per state. Combined, S4–S7 Data contain 500 unique stochastic simulations per state.
(CSV)

S5 Data Simulated infections. File containing 125 stochastic realizations of monthly infections (including both infant and noninfant infections) per state. Combined, S4–S7 Data contain 500 unique stochastic simulations per state.
(CSV)

S6 Data Simulated infections. File containing 125 stochastic realizations of monthly infections (including both infant and noninfant infections) per state. Combined, S4–S7 Data contain 500 unique stochastic simulations per state.
(CSV)

S7 Data Simulated infections. File containing 125 stochastic realizations of monthly infections (including both infant and noninfant infections) per state. Combined, S4–S7 Data contain 500 unique stochastic simulations per state.
(CSV)

S8 Data Expected infections and seasonal transmission. File containing the monthly expected number of infections per state (i.e., particle filtering mean), along with the MLEs of the seasonal transmission rate, which in our model is also the reproductive ratio.
(CSV)

S9 Data Reconstructed infections. File containing reconstructed annual infections based on particle filtering means, along with data on the number of reported cases, births, and the population size. Infections and associated data are for the contiguous states and the District of Columbia.
(CSV)

S10 Data Profile data. File containing parameter sets from the Wisconsin likelihood profile. The profiled parameters are the report rate (*ρ)* and immigration rate (*ψ*). Time units are monthly and the parameters are on the natural scale, with the exception of the B-spline coefficients.
(CSV)

S11 Data Model validation data. File containing one-step-ahead predictions of the number of monthly cases (i.e., predicted cases) for the fitted region of the data.
(CSV)

S12 Data Out-of-fit predictions data. File containing one-step-ahead predictions of the number of monthly cases (i.e., predicted cases) for the out-of-fit region of the data.
(CSV)

S13 Data Simulated cases. File containing 250 stochastic realizations of monthly reported cases per state. The simulated cases in S13 Data are from the same simulations as infections in S4–S5 Data. Note, only noninfant infections are assumed to be symptomatic and reportable, and S4–S5 Data contain both infant and noninfant infections.
(CSV)

S14 Data Simulated cases. File containing 250 stochastic realizations of monthly reported cases per state. The simulated cases in S14 Data are from the same simulations as infections in S6–S7 Data. Note, only noninfant infections are assumed to be symptomatic and reportable, and S6–S7 Data contain both infant and noninfant infections.
(CSV)

## Abbreviations

AFP: acute flaccid paralysis
CDC: Centers for Disease Control and Prevention
GPEI: Global Polio Eradication Initiative
IPV: Inactivated polio vaccine
MLE: maximum likelihood estimate
NSF: National Science Foundation
OPV: oral polio vaccine
RSV: Respiratory Syncytial Virus
WPV: wild poliovirus
