# Supplementary material for: Unraveling the Transmission Ecology of Polio
Source: PLoS Biol. 2015 Jun 19;13(6):e1002172. doi: 10.1371/journal.pbio.1002172 (PMC4474890; doi:10.1371/journal.pbio.1002172)
Supplement: S1 Text — File containing model details, extended inference methods, and results. S1 Text includes multiple figures referenced in the main text. (PDF) [file pbio.1002172.s001.pdf]

Supporting Information: Unraveling the Transmission Ecology of Polio

Micaela Martinez-Bakker, Aaron A. King, Pejman Rohani

S1 Materials & Methods

S1.1 Data

Our data consisted of monthly cases reported in the weekly US Public Health Reports and the CDC Morbidity and Mortality Weekly Report from January 1931– December 1954 for each US state and the District of Columbia. The data were first published in [1], which includes discussion of data quality during this period. Data are included in CSV files as Supporting Information. Estimates of paralytic polio reporting during this time period are 60–80%, while the reporting of non-paralytic cases varied city-to-city. Monthly state–level time series of live births from 1931–1954 were downloaded from the Vital Statistics of the United States [2]. Annual state–level population size data were collected from the Population Distribution Branch of the US Census Bureau [3].

S1.2 Data Analysis

An epidemic fadeout was defined as three consecutive months without reported cases. The portion of fadeout months was calculated for each state and each year as the number of months that were part of a fadeout, divided by the total months in the era. The relationship between the portion of fadeout months and state population size (taken as the mean population size for the era) was fit using a negative exponential to the fadeout data. A negative exponential (Eq S1) was fit to each era independently using Nonlinear Least Squares, nls, in R. The following is the equation used to relate fadeouts to population size:

$$\text{portion of fadeout months} = be^{-aN}, \qquad \text{(Eqn. S1)}$$

where N is $\log_{10}$(population size). The pairwise epidemic synchrony between states was measured using the spatial nonparametric covariance function in the R package ncf [4]. The distance between states was measured as the pairwise distance between the population center of each state. Population centers were gathered from the US Census Bureau from the 2000 US Census. Data from `https://www.census.gov/geo/reference/centersofpop.html`. Population centers were used from the year 2000 because, at the time of this project, mid-20th

century estimates of population centers were not available. However, the US Census has now made historical population centers available.

In order to test for periodicity in epidemics, a wavelet spectral analysis was conducted for each state using the R package biwavelet [5]. Significance of the 1 year period was tested using the lag-1 autocorrelation, with state-specific lag-1 autocorrelation calculated for each time series. Since the 1 year period was significant for each state, phase angle time series were constructed for the 1 year period in order to measure the seasonal timing of epidemics. For each year, the states were ranked $1 - 49$, with 1 being the state for which the epidemic peaked earliest in the year, and 49 being the state with the latest epidemic peak. The states were ranked according to their phase angle in the month of August. The phase angle is zero at the epidemic peak, negative before the peak, and positive after the peak. Thus, the state with the largest positive phase angle was ranked 1. The epidemic timing was regressed with latitude and mapped in order to test for spatial patterns.

S1.3 Model

To mechanistically model polio epidemiology we utilized a Partially Observed Markov Process (POMP) model which is suited for dealing with epidemiological data where the state variables (susceptible, infected, recovered individuals) are not observed in the data; rather the infected individuals are partially observed through case reports. For our process model we used a seasonally-forced stochastic monthly discrete-time SIR model where transitions followed a Poisson process. The model has a single recovered class that accounts for life-long immunity. The model contains 6 classes (S_i^B) of infants susceptible to infection but protected from clinical illness by maternally antibodies. The 6 susceptible infant classes contain 0–1 month olds, 1–2 month olds, etc., up to 6 month olds. The model had a single infected class for infants (I^B). The older age class contains individuals $>$ 6 months of age, and these individuals have their own susceptible (S^O) and infected class (I^O). The measurement model translated infections to clinical reported cases. We assume that infections in individuals under 6 months of age are asymptomatic, and only individuals over 6 month of age can be symptomatic and reported as a clinical case. Refer to the main text for model schematic, which was made with the R program diagrammeR (`http://rich-iannone.github.io/DiagrammeR/`) [6].

S1.3.1 Process Model

The force of infection, also referred to as the risk of infection, was

$$\lambda_t = \left(\beta_t \frac{I_t^O + I_t^B}{N_t} + \psi\right)\epsilon_t, \qquad \text{(Eqn. S2)}$$

with the following condition imposed,

$$\lambda_t = \begin{cases} \lambda_t, & \text{if } \lambda_t \geq 0 \\ 0, & \text{otherwise} \end{cases}. \qquad \text{(Eqn. S3)}$$

The first term of the force of infection represents transmission that occurs locally by individuals infected in the state at time t. Whereas, the second term, ψ, encompasses WPV that arises in the population in a way that is divorced from the local infection dynamics. These infections can include immigration of infected individuals from other geographic locations, environmental sources of WPV, and individuals shedding with an infectious period longer than a month. The transmission parameter β_t was parameterized using a B-spline with 6 basis functions and degree 2, giving it the flexibility to have either a constant transmission rate or a seasonal transmission rate,

$$\beta_t = \exp \sum_{i=1}^{6} q_i \xi_{i_t}, \qquad \text{(Eqn. S4)}$$

where each ξ_{i_t} is a periodic B-spline basis with a 1 year period. The periodic.bspline.basis() function in the pomp package was used to construct the B-spline. The process noise, ϵ_t, was gamma distributed,

$$\epsilon_t \sim \Gamma\left(\frac{1}{\Theta}, \Theta\right). \qquad \text{(Eqn. S5)}$$

The scale parameter, Θ, of the gamma distribution accounts for both environmental and demographic stochasticity,

$$\Theta = \left(\frac{\beta_{sd_1}}{\sqrt[2]{\beta_t \frac{I_t^O + I_t^B}{N_t} + \psi}} + \beta_{sd_2} \right)^2. \qquad \text{(Eqn. S6)}$$

The first term of Θ represents demographic stochasticity that is modulated by the force of infection, λ_t. Whereas, the second term represents environmental stochasticity. Formulating the process noise in this way gives us some useful properties, Eq S7–S10.

$$\mathbb{E}\left[\epsilon_t\right] = 1, \qquad \text{(Eqn. S7)}$$

and

$$\mathrm{Var}\left[\epsilon_t\right] = \Theta. \qquad \text{(Eqn. S8)}$$

Thus, the variance can capture purely environmental stochasticity, under the following parameterization:

$$\mathrm{Var}\left[\epsilon_t\right] = \beta_{sd_2}^2, \text{ if } \beta_{sd_1} = 0, \qquad \text{(Eqn. S9)}$$

and it can represent purely demographic stochasticity under alternate parameterization:

$$\mathrm{Var}\left[\epsilon_t\right] = \frac{\beta_{sd_1}^2}{\beta_t \frac{I_t^O + I_t^B}{N_t} + \psi}, \text{ if } \beta_{sd_2} = 0. \qquad \text{(Eqn. S10)}$$

Due to the computational intensity of this project, we used a discrete time model with a 1 month time step to speed-up simulation. We implemented the model as a Poisson process. There is one probability, p_{S_t}, governing the movement of individuals out of their susceptible class. p_{S_t} is the probability that a susceptible individual remains susceptible,

$$p_{S_t} = e^{-(\delta+\lambda_t)}. \qquad \text{(Eqn. S11)}$$

One minus the probability of remaining susceptible is the probability of either being infected or dying. The parameter δ is the natural death rate. The superscripts ^{B} and ^{O} will indicate infants (i.e., babies) and older individuals, respectively. The equation for the first susceptible infant class is:

$$S^B_{1_{t+1}} = B_{t+1}, \qquad \text{(Eqn. S12)}$$

where B_{t+1} are the births in month $t+1$. Similarly, the other five susceptible infant classes are tracked using the following equations:

$$S^B_{j_{t+1}} = S^B_{j-1_t} p_{S_t}, \text{ for } j \in 2:6; \qquad \text{(Eqn. S13)}$$

infants in the $j-1$ susceptible infant class at time t move into infant class j at time $t+1$ if they are not infected or die of natural death. The equation for the susceptible non-infant age group is:

$$S^O_{t+1} = \left(S^B_{6_t} + S^O_t\right) p_{S_t}. \qquad \text{(Eqn. S14)}$$

The first term of the S^O equation represents the movement of infants from the oldest infant class S^B_6 to the susceptible non-infant class. Whereas, the second term represents susceptible non-infants from time t remaining susceptible at time $t+1$. The infected infant class is tracked using the following equation:

$$I^B_{t+1} = \sum_{j=1}^{6} S^B_{j_t}(1 - p_{S_t})\frac{\lambda_t}{\delta + \lambda_t}. \qquad \text{(Eqn. S15)}$$

Infants from each of the six susceptible infant classes have a probability of being infected or dying, $1 - p_{S_t}$. The probability that an individual is infected, rather than death is $\frac{\lambda_t}{\delta+\lambda_t}$. The equation for infected non-infants is similar in structure,

$$I^O_{t+1} = S^O_t(1 - p_{S_t})\frac{\lambda_t}{\delta + \lambda_t}. \qquad \text{(Eqn. S16)}$$

Note, infected individuals are infected for exactly 1 month, which is the typical duration of shedding [7]. There was one rounding condition imposed on infections in the process model. If I^B_t or I^O_t were values between 0 and 1, they were rounded to 0 or 1. This was in order to prevent fractions of infected individuals.

S1.3.2 Measurement Model

In order to model the stochastic process of infected individuals becoming symptomatic and subsequently being reported as a case, we drew cases from a normal distribution,

$$\text{cases}_t \sim \text{normal}(\rho_t I_t^O, \tau I_t^O), \qquad \text{(Eqn. S17)}$$

with the following condition imposed,

$$\text{cases}_t = \begin{cases} \text{round}(\text{cases}_t), & \text{if cases}_t \geq 0 \\ 0, & \text{otherwise} \end{cases} \qquad \text{(Eqn. S18)}$$

For calculating likelihood we used a binned-normal probability density.

If $\text{cases}_t > 0$,

$$\text{Likelihood}_t = \text{pnormal}(\text{cases}_t + 0.5, \rho_t I_t^O, \tau I_t^O) - \text{pnormal}(\text{cases}_t - 0.5, \rho_t I_t^O, \tau I_t^O) + 1e^{-18}. \qquad \text{(Eqn. S19)}$$

If $\text{cases}_t = 0$,

$$\text{Likelihood}_t = \text{pnormal}(\text{cases}_t + 0.5, \rho_t I_t^O, \tau I_t^O) + 1e^{-18}. \qquad \text{(Eqn. S20)}$$

The report rate, ρ_t, was estimated for the Pre-Baby Boom era and the Baby Boom era, with the change in report rate occurring in January 1946, the start of the Baby Boom. The parameter τ captures the variation in the process of observing infections via reported cases. The scaler $1e^{-18}$ was used for practical purposes to put a lower bound on the likelihood in order to ensure finite log-likelihood values. Initially we tried using a continuous normal measurement model, however, we discovered that a continuous normal model over-inflated the likelihood of parameter sets that produced zero cases in trough months, making it difficult to identify parameter regimes that could capture both the epidemic cases and the trough cases. Thus, we used the binned-normal distribution.

S1.4 Statistical Inference

We fit our SIR model to the data from each state independently using Maximization by Iterated particle Filtering (MIF) in the R package pomp [8, 9]. pomp tutorials and vignettes may be found at `http://pomp.r-forge.r-project.org/`. MIF is a simulation-based likelihood method for parameter estimation. The basis of MIF is particle filtering, which integrates state variables of a stochastic system and estimates the likelihood for fixed parameters. Instead of fixing parameters, MIF varies them throughout the filtering process and selectively propagates particles (i.e., parameter sets) that have the highest likelihood. By initializing MIF throughout parameter space we estimated the shape of the likelihood surface for each US state and

identified the Maximum Likelihood parameter Estimates (MLEs). MIF was initialized from 1 million parameter sets for a global search, followed by additional phases of increasingly localized searches, which included profiling. In total, for each US state, MIF was initialized from tens-of-thousands of locations in parameter space to estimate the shape of the likelihood surface and identify the MLEs. We characterized seasonal transmission using a B-spline with six bases. Although the transmission rate fluctuated seasonally, there was no inter–annual variation in transmission in our model. In order to account for improved reporting brought about by the Foundation for Infantile Paralysis, we estimated two report rates, one for the Pre-Baby Boom Era and another for the Baby Boom Era. Inference was done using the data from May 1932 to January 1953, with the exception of South Dakota and Texas, whose data began later. For South Dakota and Texas, inference was done using data beginning in May 1933 and 1934, respectively.

For each state, inference was initiated in May of the year following the first full year of available data on polio cases and births. This was done in order to construct the infant initial conditions directly from the birth data. The month of May is the tail-end of the polio off-season. Thus, the 0-6 month old infants in May were born between November and April, when polio transmission was low and they were unlikely to be infected. We assumed that the initial number of susceptible infants in the 6th infant class was the number of individuals born in November of the previous year; individuals born in December were in the 5th infant class, January births were in the the 4th infant class, February births in the 3rd infant class, March births in the 2nd infant class, and April births in the the 1st infant class. We set the initial number of infected infants to zero. Constructing the infant initial conditions prevented us from having to estimate an addition seven parameters.

S1.4.1 Phase I: Global Search

To initialize a global search of parameter space, 1 million parameter combinations, with initial conditions reflecting a low incidence month, were generated to cover the range of parameter values given in Table S1. For each parameter set, ten replicate particle filters were run, each with 2000 particles, to estimate the likelihood of the data from the state of Illinois. Illinois was chosen because of the high incidence of polio in Illinois and because Illinois had an increase in incidence following the World War II Baby Boom, which was a characteristic feature of most US states. The reporting transition was fixed to January 1946, the start of the Baby Boom era, and about the time when non-paralytic polio cases started being reported. Each parameter set was placed in combination with an increase in report rate of 0% and 1%. The 1 million likelihoods were used to provide a rough estimate of the global shape of the likelihood surface and identify regions of parameter space with high likelihood. Of the 1 million parameter sets, the 50000 with the highest likelihoods were used to initialize Phase II. It is important to note that the particle filter gives an unbiased estimate of the likelihood, but some extra algebra needs to be done to get the estimates of the log-likelihood from replicate particle filters. We calculated the unbiased estimate of the log-likelihood using Eqns. S21–S24.

Table S1: Parameter Bounds used in Global Search. Values in the table are on the natural scale, rates are given in months. ρ is the report rate for the Pre-Baby Boom era, and $\Delta\rho$ is the increase in report rate during the Baby Boom. The natural death rate δ was fixed. The search scale indicates whether parameters were transformed into the log or logit scale for searching parameter space. The transformation ensured the breadth of the search. Note, that parameter searches were not restricted to these bounds, rather these bounds were used to initialize searches for parameter space.

parameter	interpretation	lower bound	upper bound	search scale
ρ	report rate	0.0001074816	0.9999401	logit
$\Delta\rho$	increase in report rate	0	0.01	logit
τ	measurement stochasticity	1.024683e-06	2390.506	log
ψ	external source of infection	1.843172e-07	114.648	log
δ	natural death rate	0.001388889	NA	NA
β_{sd_1}	demographic stochasticity	1.835635e-07	21.59023	log
β_{sd_2}	environmental stochasticity	1.217956e-07	15.25643	log
q_i	B-spline basis coefficients	9.152956e-12	770910.8	log

$$\log \vec{\mathcal{L}}(\Theta) = \vec{\mathbf{x}}. \qquad \text{(Eqn. S21)}$$

Given the vector of log-likelihoods, $\vec{\mathbf{x}}$, produced from n replicate particle filters under the parameter set Θ (not to be confused with Θ in Eq. S5), we find the mean,

$$y = \mathsf{mean}(\vec{\mathbf{x}}). \qquad \text{(Eqn. S22)}$$

We used the mean to estimate the log-likelihood,

$$\log \mathcal{L} = y + \log(\mathsf{mean}(e^{\vec{\mathbf{x}}-y})), \qquad \text{(Eqn. S23)}$$

and calculate the standard error of the log-likelihood,

$$\log \mathcal{L}_{\mathsf{se}} = \frac{\mathsf{sd}(e^{\vec{\mathbf{x}}-y})}{\sqrt{n}} \frac{1}{e^{\log \mathcal{L}-y}}, \qquad \text{(Eqn. S24)}$$

where sd is the standard deviation.

S1.4.2 Phase II: Initial Condition Improvement

Each of the 50000 parameter sets obtained from Phase I was taken in pairwise combination with the data from each of the 49 continental US states. For each parameter set and US state combination, MIF was used to improve the estimates of the initial conditions using the first 12 months of data. Each initial condition had a random walk standard deviation of 0.02. Each

MIF had 45 iterations, 2000 particles, hyperbolic cooling with a variance factor of 2, and a cooling fraction of 0.5. This resulted in 50000 parameter sets for each state, each with unique initial conditions. For each parameter set, ten replicate particle filters were run, each with 2000 particles, to estimate the likelihood of the full time series (i.e., the fitted region of the data) from the state. All searches of parameter space were done on the log or logit scale. The log scale was used to enforce parameter values > 0 and the logit scale was used to bound parameters, such as the report rate, between 0 and 1. See Equations below for expit and logit functions.

$$\text{logit}(p) = \log\left(\frac{p}{1-p}\right) \qquad \text{(Eqn. S25)}$$

$$\text{expit}(x) = \frac{1}{1+e^{-x}} \qquad \text{(Eqn. S26)}$$

S1.4.3 Phase III–V: Local Searches of Parameter Space

For each US state, the 25000 highest-likelihood parameter sets from Phase II were carried forward into Phase III. Therefore, in Phase III, MIF was initiated from a total of 1225000 parameter sets. Each initial condition and all other estimated parameter were allowed to vary in MIF with a random walk standard deviation of 0.02, with the exception of $\Delta\rho$ which was given a random walk standard deviation of 0.03. It is important to note that the random walk standard deviations for ρ and $\Delta\rho$ were time-varying. ρ was estimated using the data up through December 1945, and $\Delta\rho$ was estimated using the data beginning in January 1946. Thus, the random walk standard deviation for ρ was zero from Jan 1946 onward. Likewise, the random walk standard deviation for $\Delta\rho$ was zero up until January 1946. In order to define time-varying random walk standard deviations, the MIF2 method was used in the developer version of pomp, version 0.44-1. Refer to Table S1 for a list of estimated parameters. Each MIF run had 45 iterations, 2000 particles, hyperbolic cooling with a variance factor of 2, and a cooling fraction of 0.5. The reporting transition remained fixed at January 1946, although $\Delta\rho$ was allowed to vary. After running MIF, to estimate the likelihood using each new parameter set, ten replicate particle filters were run, each with 2000 particles.

In Phase IV, the 1225000 parameter sets from Phase III were culled to remove all parameter combinations that had a likelihood 20 log-likelihood units below the maximum for that state. Using the culled parameter sets, MIF was run with 45 iterations, 2000 particles, hyperbolic cooling with a variance factor of 2, and a cooling fraction of 0.5. Random walk standard deviations were the same as Phase III. To estimate the likelihood, ten replicate particle filters were run, each with 2000 particles.

In Phase V, 49000 parameter sets were chosen using the 1000 top likelihood parameter sets for each US state from Phase IV. In Phase V, MIF was run with 45 iterations for each

parameter set using the same MIF settings as Phase IV, with the exception of the $\Delta\rho$ random walk standard deviation, which was 0.02.

S1.4.4 Phase VI & VII: Profiling Report Rate and Immigration

We found the range of ρ and ψ in the top parameter sets for each state. For profiling in Phase VI, ρ and ψ values were generated to span the range. The ranges were [0.001, 0.1] and [6e-6, 2.5e-3], for ρ and ψ, respectively. There were 37 values of ρ, $(\rho_1, \rho_2, ..., \rho_{37})$, with 0.001 interval sampling in the range of [0.001, 0.03] and 0.01 interval sampling in the range of [0.04, 0.1]. There were 50 values of ψ, $(\psi_1, \psi_2, ..., \psi_{50})$ evenly sampled across the range. In order to make the profiles two dimensional, we took every pairwise combination of ρ and ψ, which resulted in 1850 profile points, each being a unique combination of ρ and ψ. Next, we needed to identify the other parameters to join with our 1850 combinations of ρ and ψ. At each of the 1850 profile points we used four parameter set variants for each US state. The first variant was the ρ_i and ψ_j combination substituted into the maximum likelihood parameter set from Phase V (i.e., the US state-specific maximum). The second variant was the ρ_i and ψ_j combination substituted into the highest likelihood parameter set for which $\rho_{i-1} \leq \rho \leq \rho_{i+1}$. The third was the ρ_i and ψ_j combination substituted into the highest likelihood parameter set for which $\psi_{j-1} \leq \psi \leq \psi_{j+1}$. The fourth was the ρ_i and ψ_j combination substituted into the parameter set with the minimum Euclidean distance from ρ_i and ψ_j.

With four parameter set variants at each of the 1850 profile values, there were a total of 7400 parameter sets used to initialize the 2-D profile for each state. ρ and ψ were fixed at the given profile values, and MIF was used to maximize the likelihood along the other parameter and initial condition dimensions. Starting at each of the 7400 parameter set variants, two replicates of MIF were run. Each estimated parameter and initial condition had a random walk standard deviation of 0.02. Once again, the random walk standard deviations for ρ and $\Delta\rho$ were time-varying. There were 45 iterations of MIF per replicate, with 2000 particles, hyperbolic cooling with a variance factor of 2, and a cooling fraction of 0.5. The reporting transition remained fixed at January 1946. This resulted in 14800 parameter sets for each state, and 725200 parameter sets across all US states. For each state, the likelihood of the 14800 parameter sets was estimated by running 10 replicate particle filters, each with 2000 particles.

In Phase VII, we found the highest likelihood parameter set at each state's 1850 profile points. The ρ and ψ values remained fixed at each profile point and two replicate MIF runs were preformed. Now each estimated parameter and initial condition had a random walk standard deviation of 0.01.There were 60 iterations of MIF per replicate, with 4000 particles, hyperbolic cooling with a variance factor of 2, and a cooling fraction of 0.5. This resulted in 3700 parameter sets for each state, and 181300 parameter sets across all US states. For each state, the likelihood of the 3700 parameter sets was estimated by running 10 replicate particle filters, each with 4000 particles.

S1.4.5 Phase VIII: Profiling Report Rate and Immigration with Constant Reporting Assumption

In order to test whether the report rate increased during the Baby Boom era, we repeated the profiling in Phase VII, with $\Delta\rho = 0$. For each US state we then used AIC to determine which model best fit the data, the model with $\Delta\rho = 0$ or the model with $\Delta\rho > 0$. For each state, the MLE was taken from the model with the lower AIC.

S2 Results

S2.1 Patterns in the Data

The negative exponential functions representing the relationship between fadeouts and population size for the Pre-Baby Boom and Baby Boom eras are given in Eqns S27–S28.

$$\text{portion of fadeout months}_{\text{Pre-Baby Boom}} = 9195e^{-1.8N} \mid N \text{ is } \log_{10}(\text{population size}) \quad \text{(Eqn. S27)}$$

$$\text{portion of fadeout months}_{\text{Baby Boom}} = 10230e^{-2N} \quad \text{(Eqn. S28)}$$

S2.2 Inference Results

Model Validation. We used the MLE for each state to calculate the expected number of infected non-infants for each month of the fitted data. The expected number of infections was obtained by running a particle filter with 2000 particles and taking the prediction mean of the particles at each time point. The prediction mean is the expected state value at time t, given the data and the state values up to time $t-1$.

$$\mathbb{E}(\mathbf{X}_t \mid \mathbf{X}_{t-1}, \text{cases}_{t-1}). \quad \text{(Eqn. S29)}$$

In order to get the expected number of cases, we took the expected number of non-infant infections and multiplied it by the state- and time-specific report rate,

$$\mathbb{E}(\text{cases}_t) = \rho_t \mathbb{E}(I^O_t \mid \mathbf{X}_{t-1}, \text{cases}_{t-1}). \quad \text{(Eqn. S30)}$$

For model validation, the r-squared was quantified for the model as the reduction in the sum-of-squared deviations obtained when using the expected cases from the state-specific MLE versus a state-specific null model of the mean number of cases.

$$R^2 = 1 - \frac{\sum_t \left[\mathbb{E}(\text{cases}_t) - \text{cases}_t\right]^2}{\sum_t \left[\text{mean}(\text{cases}) - \text{cases}_t\right]^2}. \quad \text{(Eqn. S31)}$$

The generalized r-squared was quantified for the model as the reduction in the sum-of-squared deviations across all US states obtained when using the expected cases from the state-specific

MLE versus a state-specific null model of the mean number of cases.

$$R^2_{\text{generalized}} = 1 - \frac{\sum_{i=1}^{49} \sum_t \left[\mathbb{E}(\text{cases}_t) - \text{cases}_t\right]^2}{\sum_{i=1}^{49} \sum_t \left[\text{mean}(\text{cases}) - \text{cases}_t\right]^2}. \quad \text{(Eqn. S32)}$$

The out-of-fit predictions were done using the same procedure as was used for the fitted data region. The out-of-fit predictions were done using the data from the last two epidemic years, January 1953–December 1954.

Predicated Latitudinal Gradient. In order to determine whether or not the model reproduces the latitudinal gradient in the timing of polio epidemics, 10 stochastic simulations were run using the MLE for each state. For these 10 simulations, we used the inference time period inclusive to all states, May 1934–January 1953. The simulations were taken together to test for a gradient in the simulated data. A wavelet analysis was run on the simulated time series and the phase angles associated with the 1 year period were used to rank the states 1-49, 1 being the earliest epidemic peak and 49 being the latest epidemic peak. We found that the model reproduces the latitudinal gradient in the timing of polio epidemics (main text Fig 3) with epidemics peaking earlier in the southern US and later in the northern US (observed gradient shown in Fig S1). In order to determine whether the latitudinal gradient in the timing of polio epidemics is driven by the latitudinal gradient in birth peak timing, and/or seasonal birth amplitude, we simulated the model with birth seasonality removed. We did this by decomposing the birth time series using the stl() function in the stats package in R and using the trend in births as a model covariate, rather than using the actual births. We found that the model without birth seasonality also displays a latitudinal gradient (Fig S5). The latitudinal gradient in the timing of polio epidemics is largely driven by the latitudinal variation in the timing of peak polio transmission. Fig S7 shows that the peak transmission rate for polio occurs between May and July in the US. The peak transmission rate in southern states typically occurs in May and the peak transmission rate in northern states occurs in July, a couple months earlier than their respective peaks in incidence. In order to test whether the predicted timing of peak polio incidence matches the observed timing, we compared the mean rank calculated from the data and the mean rank based off simulation. See main text Fig 3D, showing the correlation between the observed and predicted mean rank. You will also see in Fig S5C–D that the simulated slope and R^2 values of latitude vs. mean rank reflect a latitudinal gradient.

While geographical variation in birth seasonality was insufficient to explain the latitudinal gradient seen in epidemic timing, birth seasonality played a role in shaping seasonal incidence of polio. Epidemiological theory indicates that birth seasonality can have important dynamical consequences for childhood diseases [10, 11, 12]. To quantify the influence of birth seasonality (Fig S4) on infant infections, we compared simulations of the fitted models to simulations for which seasonal fluctuations in births were removed. In the presence of birth seasonality, infant infection incidence was generally higher during the epidemic peak (Fig S6); however,

since infant infections were assumed to be asymptomatic, this did not affect the incidence of disease directly, and no indirect effect was observed. We attribute the negligible effect of birth seasonality on disease incidence to the low amplitude of birth seasonality, which was $\sim 10\%$ in the US at this time[11].

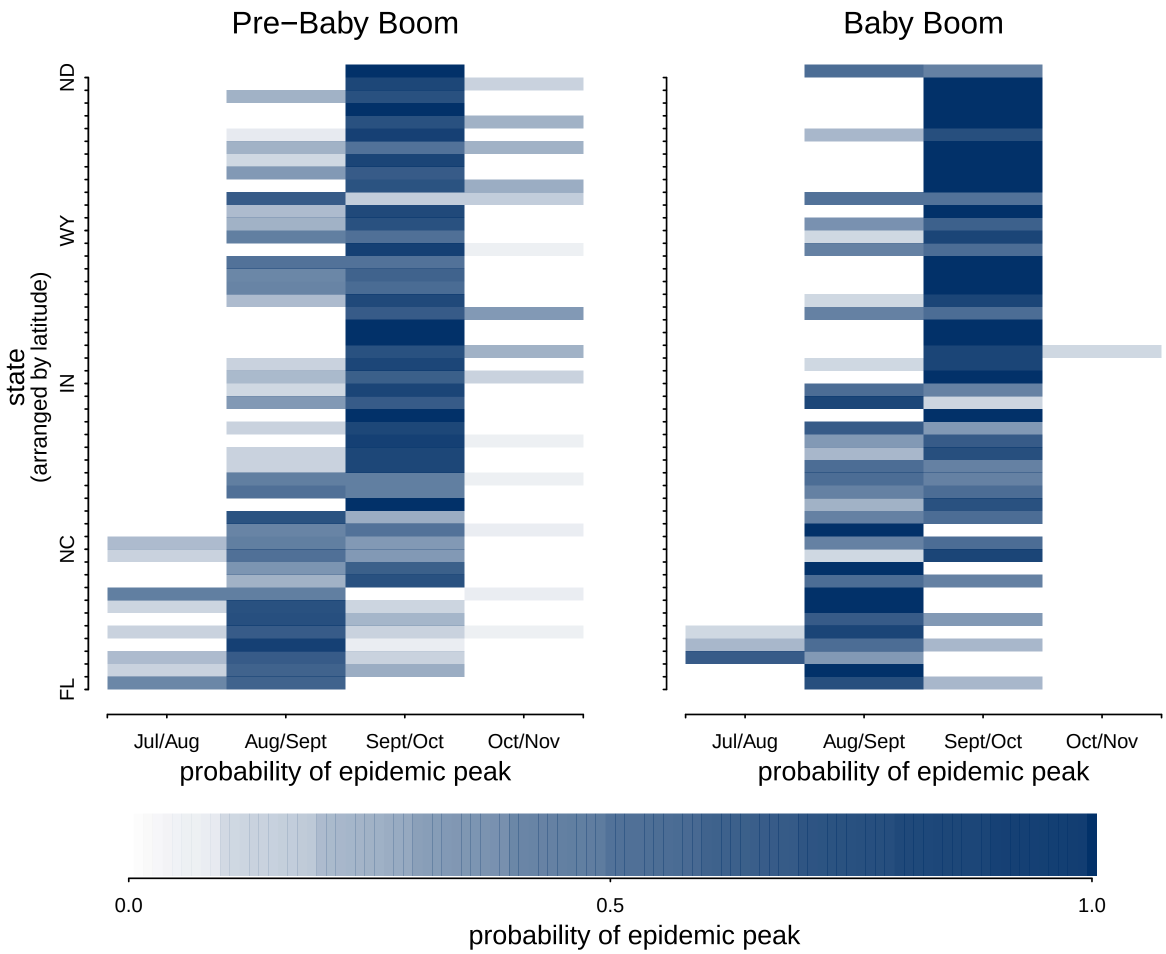

Figure S1: Probability densities of the timing of the epidemic peak for each state during the Pre-Baby Boom and Baby Boom eras. For each state, the timing of the epidemic peak was determined for each year with 20+ polio cases. The probability was measured as the portion of years (in each era) for which the peak occurred between July/Aug, Aug/Sept, Sept/Oct, and Oct/Nov. Peak timing was measured using the 1 yr wavelet band phase angle.

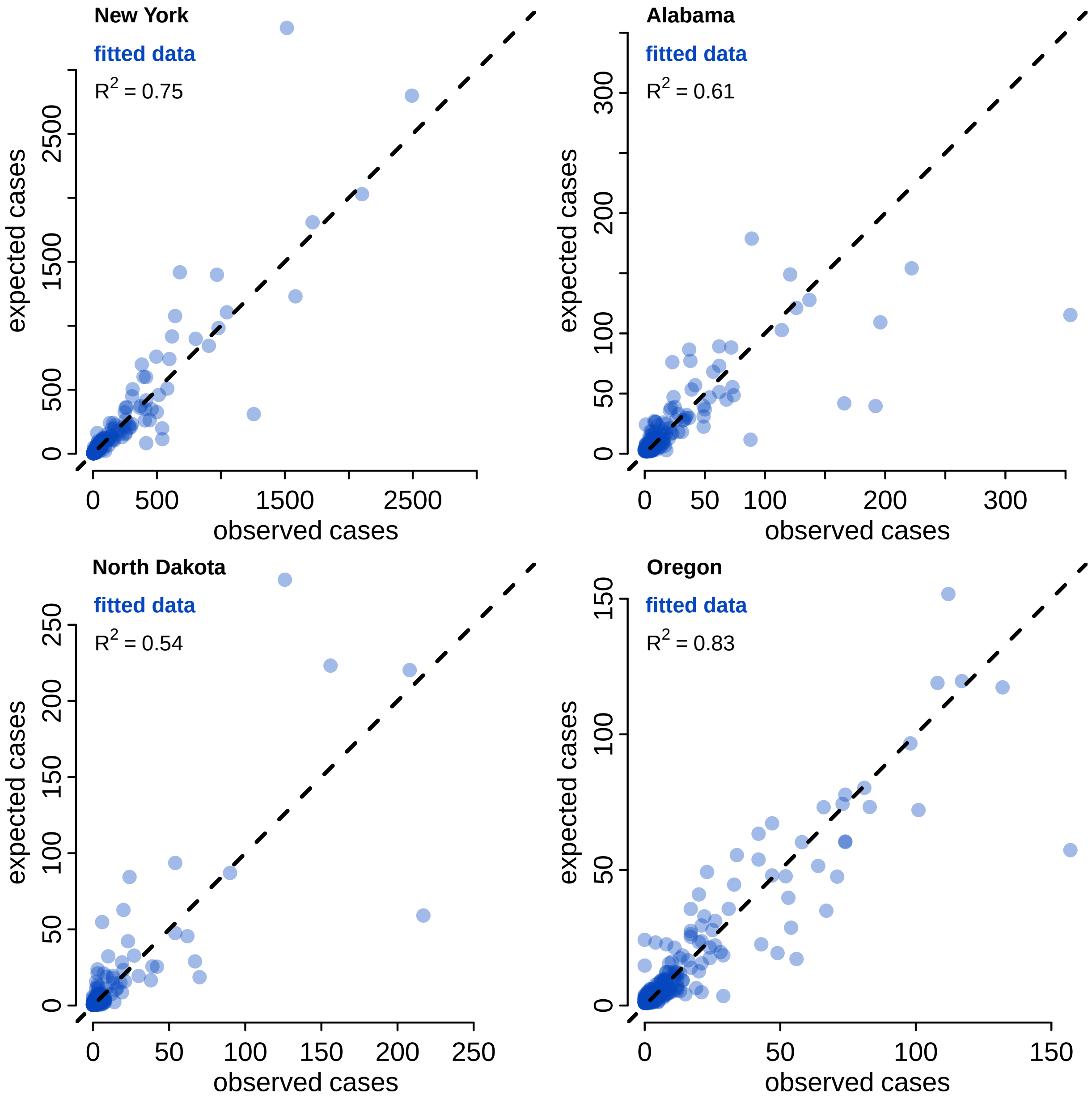

Figure S2: One-step-ahead predictions based on the MLE model for each of four states. Observed and expected cases are shown on the natural scale, in contrast to Fig 3B in the main text, which is on the $\log_{10}$ scale. The R^2 was calculated as shown in Eq. S31.

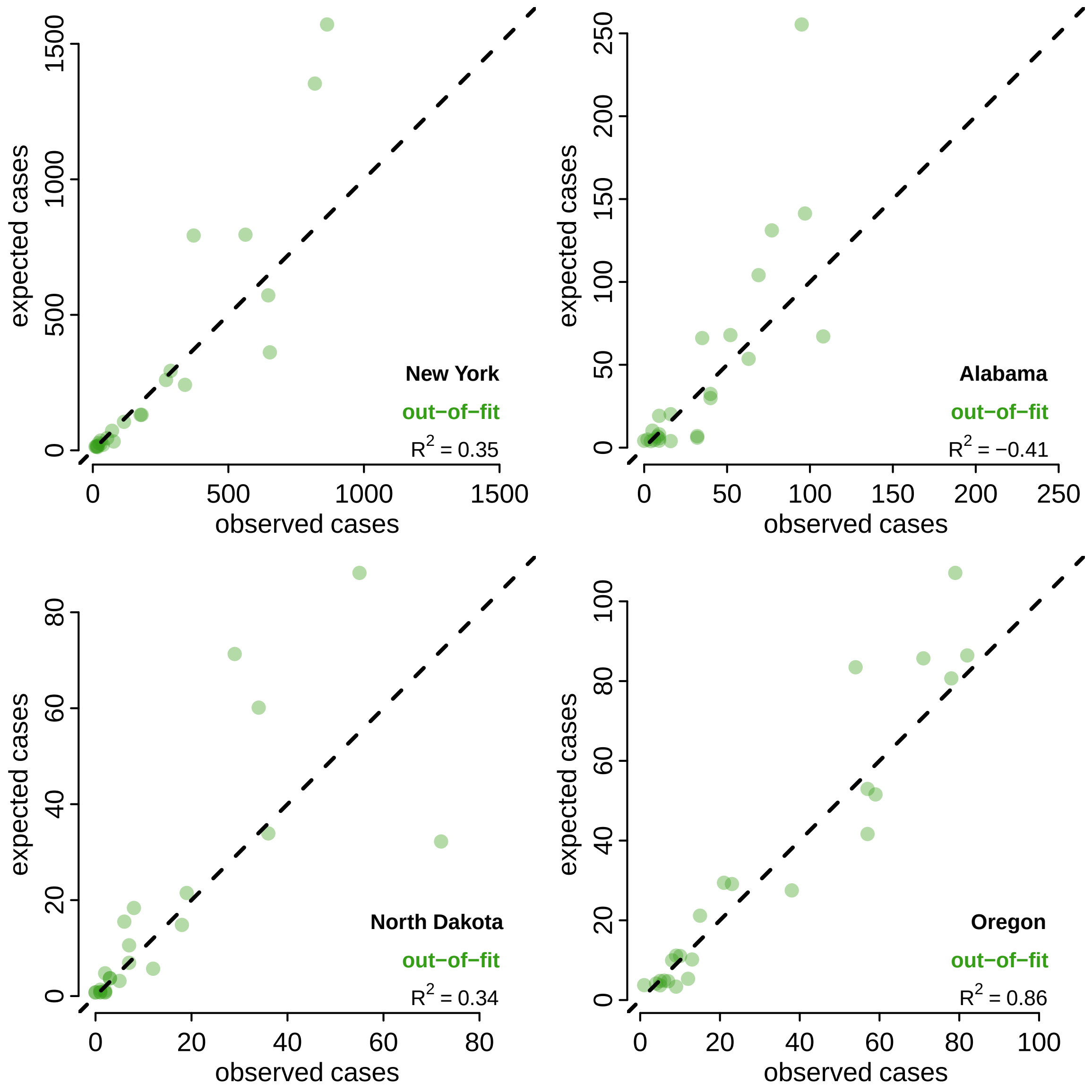

Figure S3: Out-of-fit predictions based on the MLE model for each of four states. Observed and expected cases are shown on the natural scale, in contrast to Fig 3C in the main text, which is on the $\log_{10}$ scale. The R^2 was calculated as shown in Eq. S31. A negative R^2 value indicates that the null model had a lower sum of squared deviations than the fitted model for the out-of-fit predictions, which was the case for 3 of the 49 states (Alabama, Connecticut, and Delaware). Alabama was shown as an example of one of the "worst-fit" states.

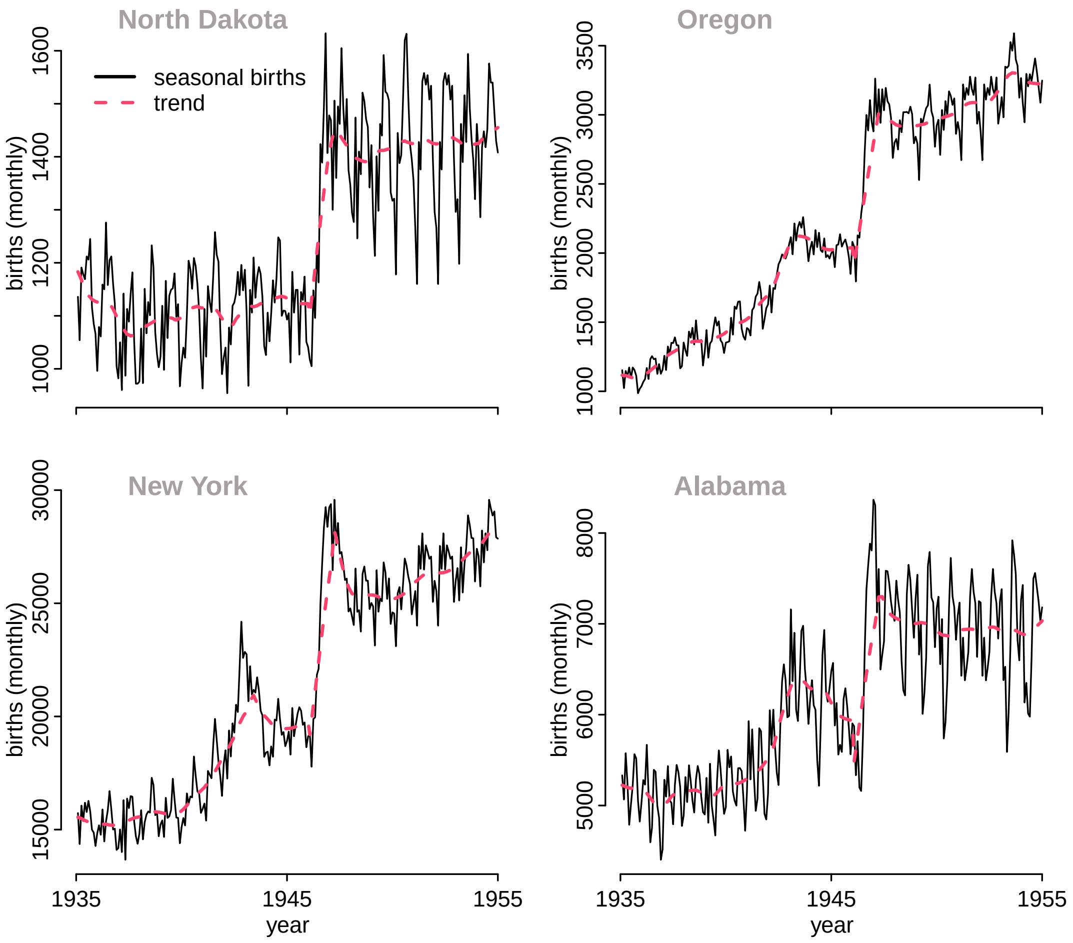

Figure S4: Monthly births (black time series) are seasonal. Monthly births were used as covariates for the fitted models. Th trend in births is shown by the dashed line (fuchsia). Four states from different geographic regions are shown.

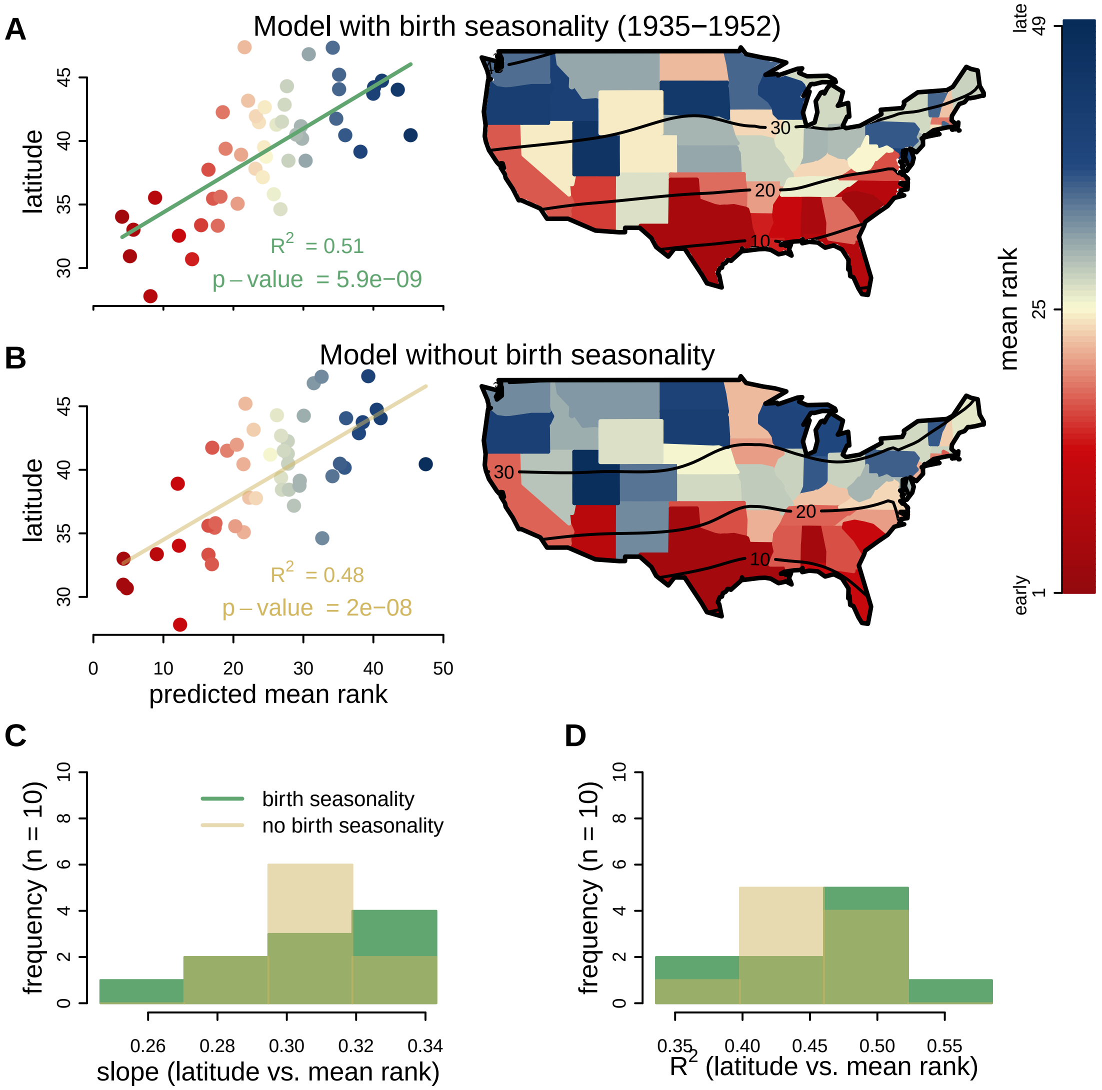

Figure S5: Latitudinal gradient predicted from model simulations using maximum likelihood parameter estimates for each state. (A) Latitudinal gradient in simulations of the fitted models with seasonal births. (B) Latitudinal gradient in simulations with birth seasonality removed (i.e., the trend in births was used). (C) The distribution of the latitudinal gradient slopes for 10 simulations for each US state for the model with birth seasonality (i.e., using the raw birth data) and the model with birth seasonality removed (i.e., using the trend in births). (D) The R^2 for the latitudinal gradient for 10 simulations with and without birth seasonality.

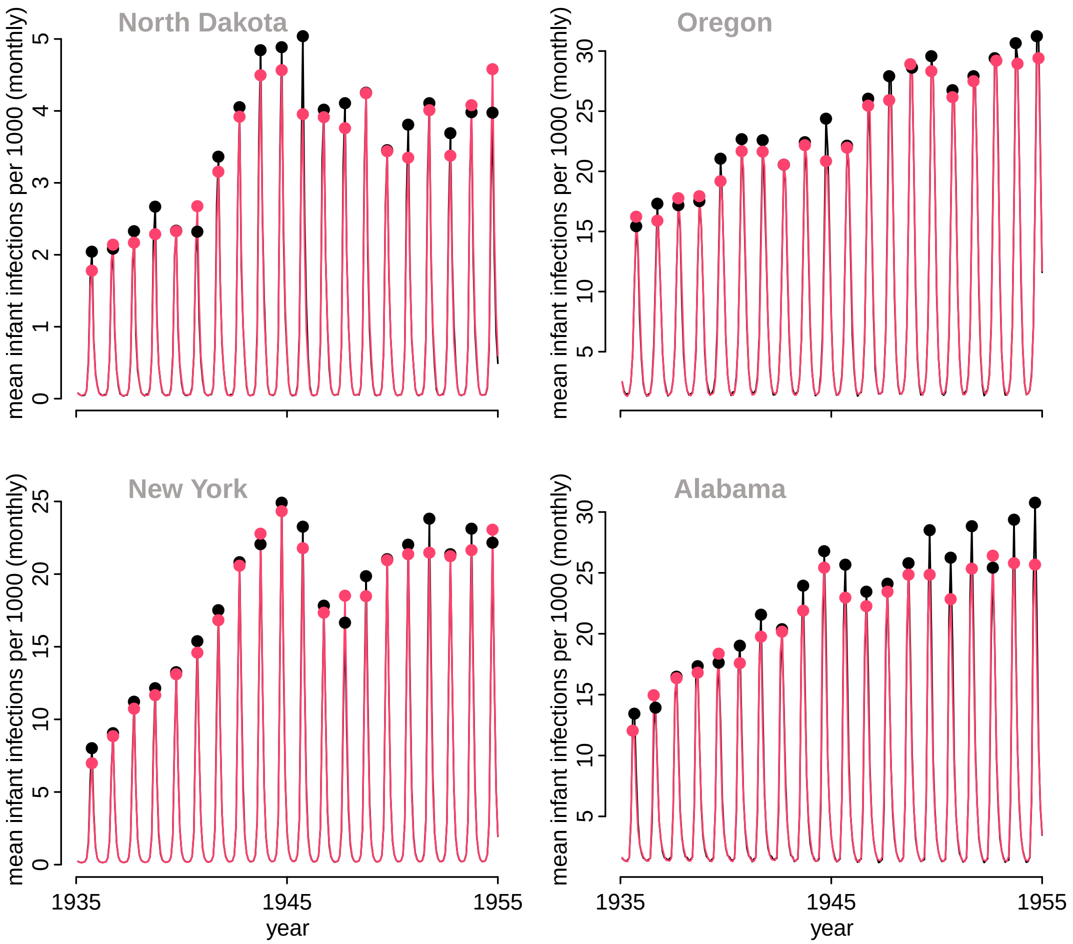

Figure S6: Mean monthly incidence of infant-infections per 1000 infants, with and without birth seasonality. Model with birth seasonality is shown in black, model without birth seasonality in fuchsia. 500 simulation were run for each state and model combination. Points indicate annual peak incidence. The model with birth seasonality generally displayed higher peak infant-infections. Four states from different geographic regions are shown.

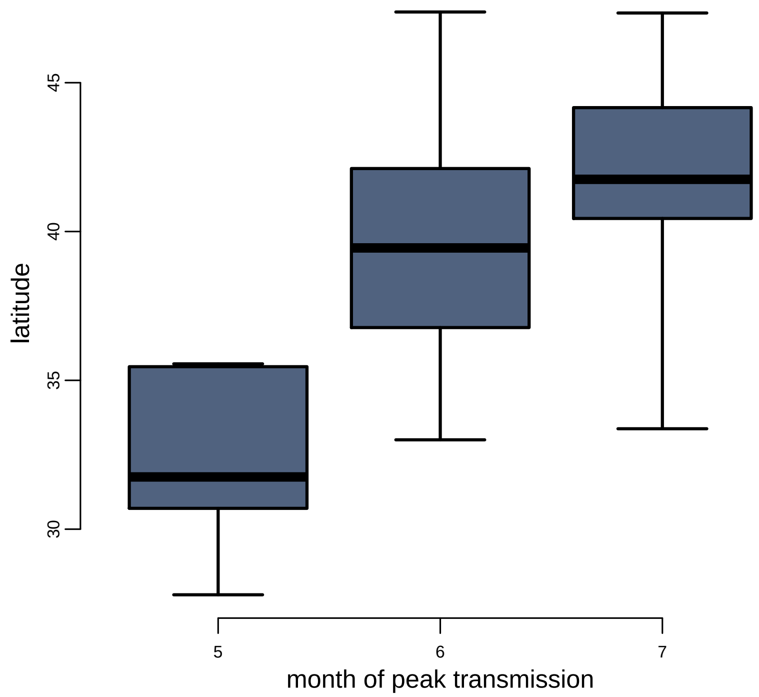

Figure S7: Latitudinal variation in MLEs of peak transmission timing.

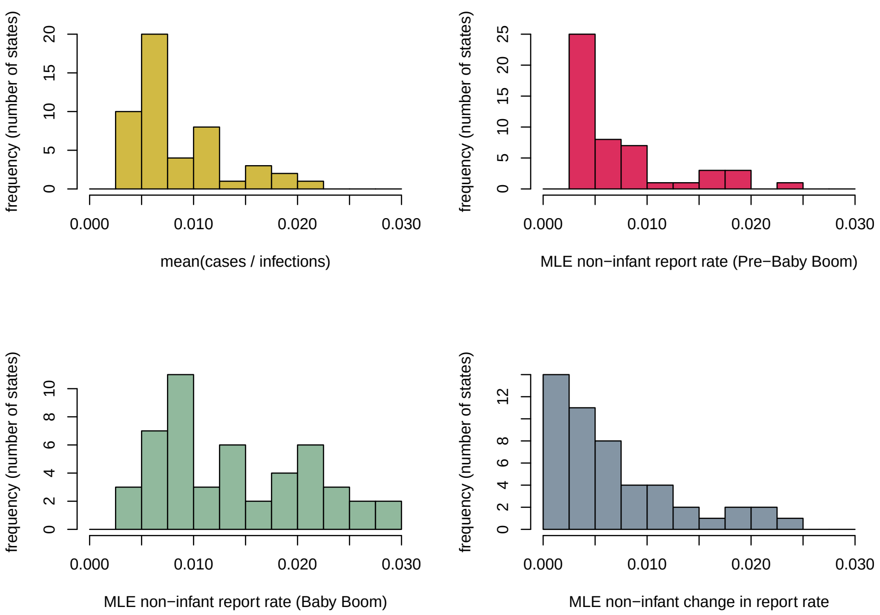

Figure S8: MLE report rates. The report rate is a composite parameter that encompasses the probability of an infected individual becoming symptomatic, and the subsequent probability that symptomatic infections are reported. (top-left) The distribution of mean report rates for infants and non-infants across states. Non-infant report rates during the Pre-Baby Boom era (top-right) were lower than that of the Baby Boom era (bottom-left), due to the increase shown in (bottom-right).

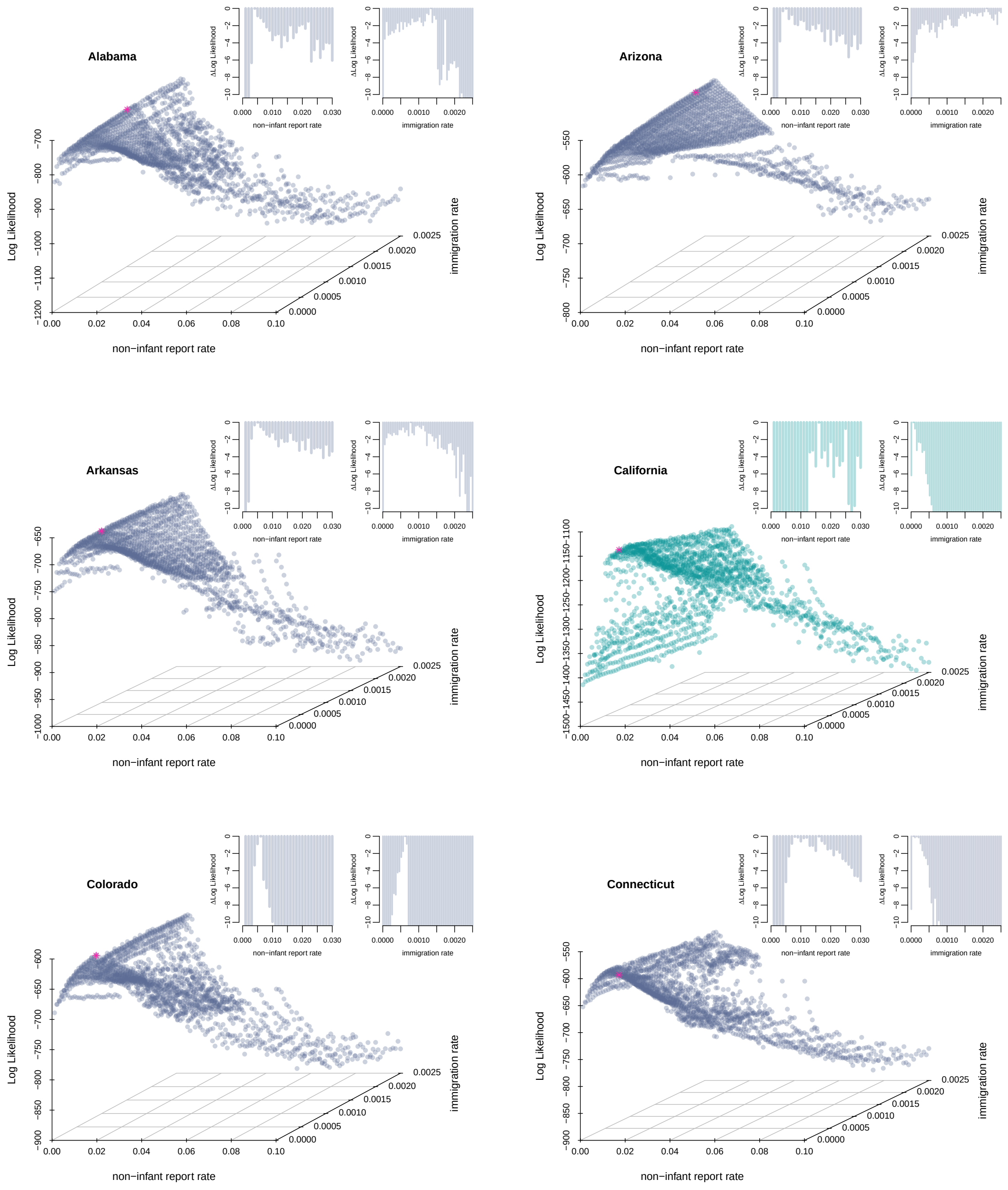

Figure S9: Likelihood profiles for the Pre-Baby Boom non-infant report rate (ρ_t) and the immigration rate (ψ). MLE indicated by pink asterisk. Profile color indicates whether the report rate was increased during the Baby Boom (purple profiles) or if the report rate was constant through time (green profiles).

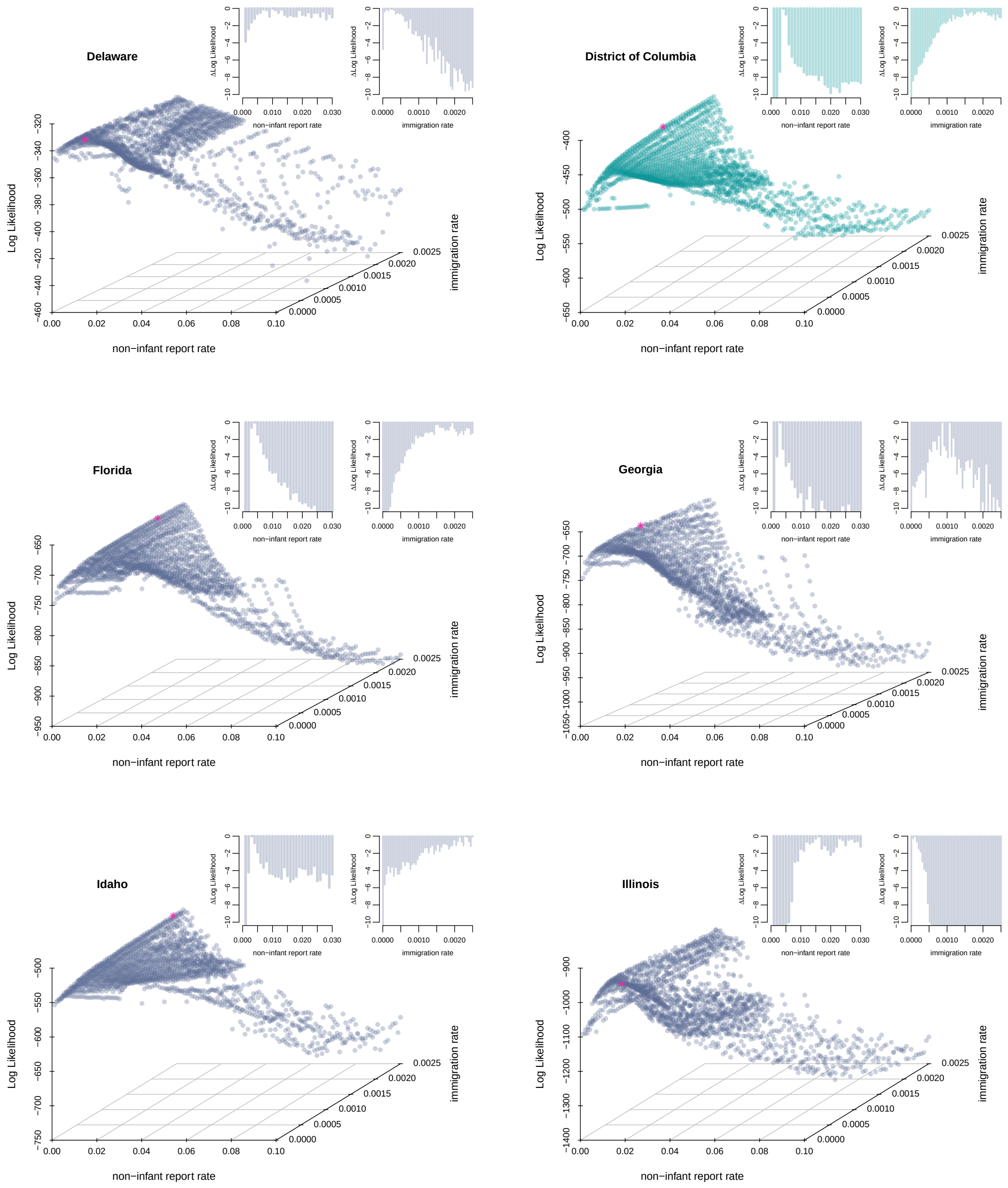

Figure S10: Likelihood profiles for the Pre-Baby Boom non-infant report rate (ρ_t) and the immigration rate (ψ). MLE indicated by pink asterisk. Profile color indicates the whether the report rate was increased during the Baby Boom (purple) or if the report rate was constant through time (green).

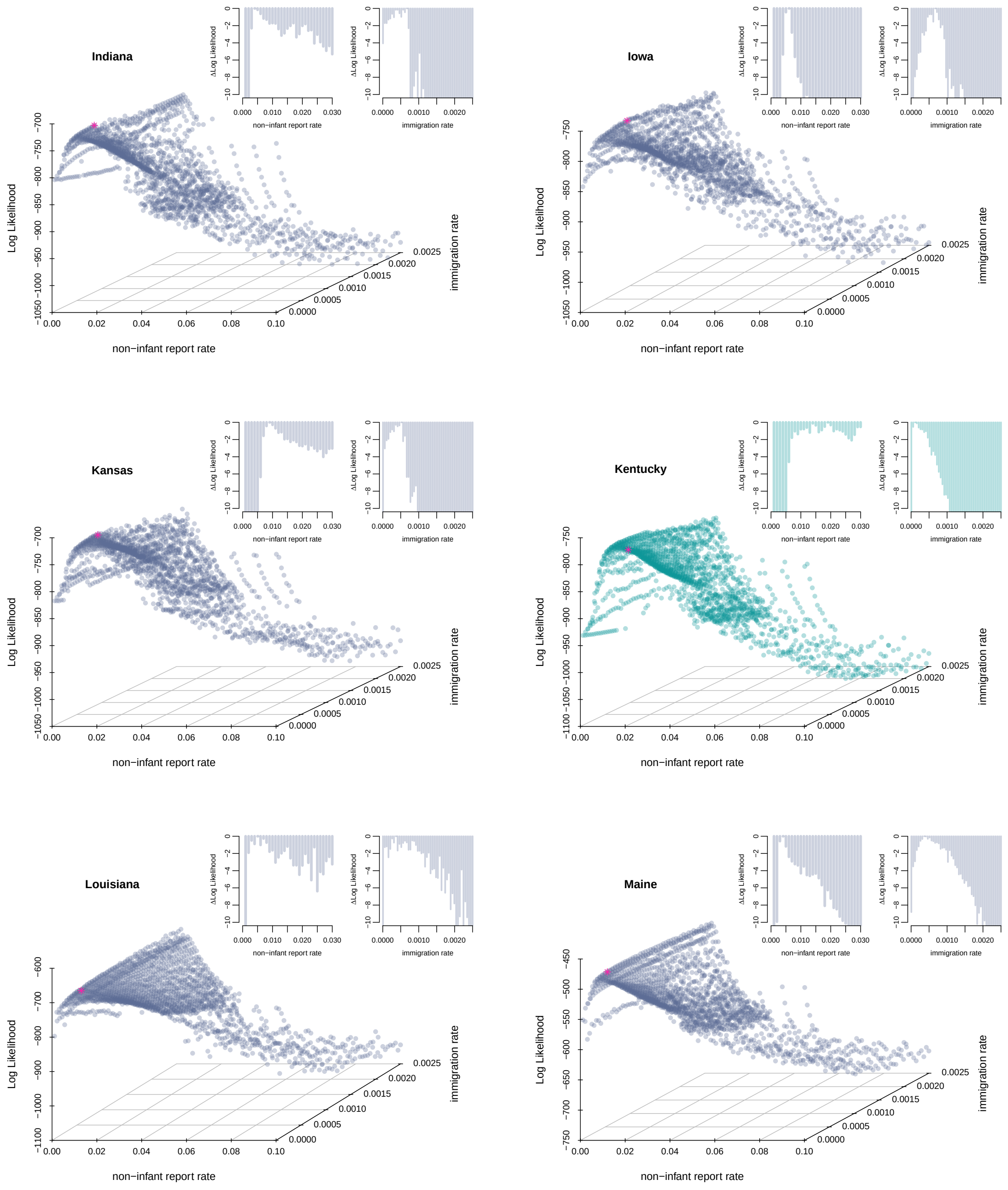

Figure S11: Likelihood profiles for the Pre-Baby Boom non-infant report rate (ρ_t) and the immigration rate (ψ). MLE indicated by pink asterisk. Profile color indicates the whether the report rate was increased during the Baby Boom (purple) or if the report rate was constant through time (green).

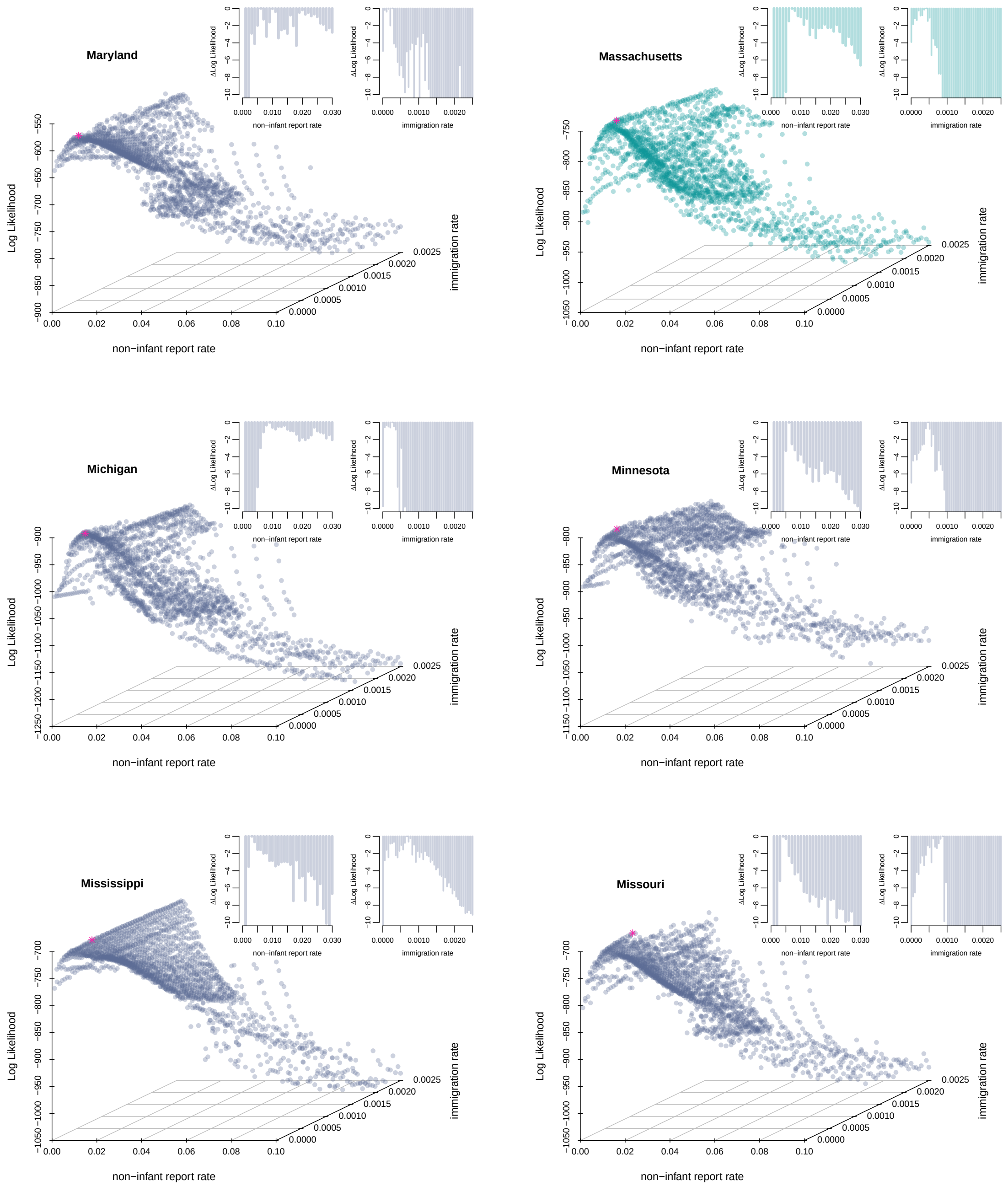

Figure S12: Likelihood profiles for the Pre-Baby Boom non-infant report rate (ρ_t) and the immigration rate (ψ). MLE indicated by pink asterisk. Profile color indicates the whether the report rate was increased during the Baby Boom (purple) or if the report rate was constant through time (green).

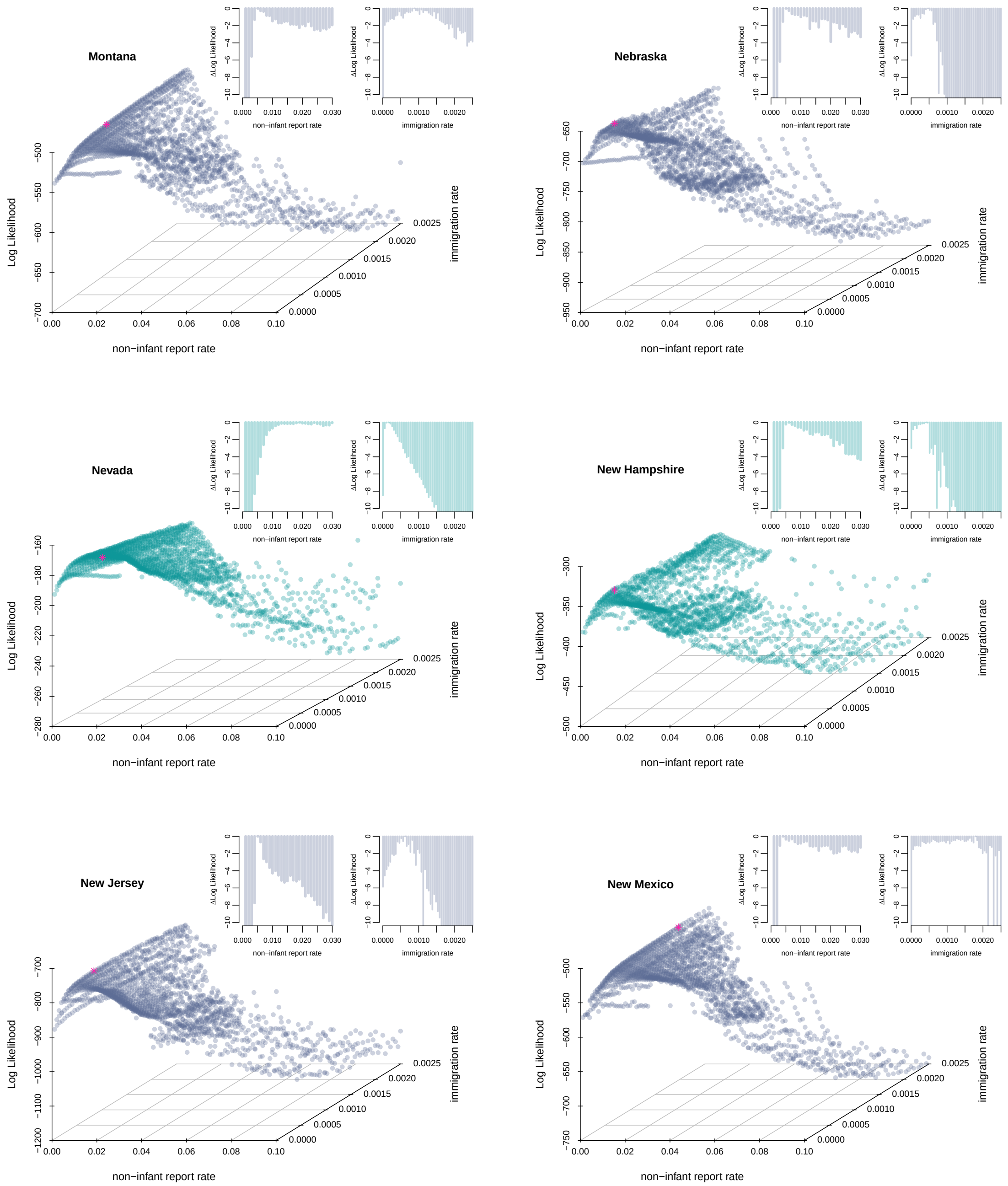

Figure S13: Likelihood profiles for the Pre-Baby Boom non-infant report rate (ρ_t) and the immigration rate (ψ). MLE indicated by pink asterisk. Profile color indicates the whether the report rate was increased during the Baby Boom (purple) or if the report rate was constant through time (green).

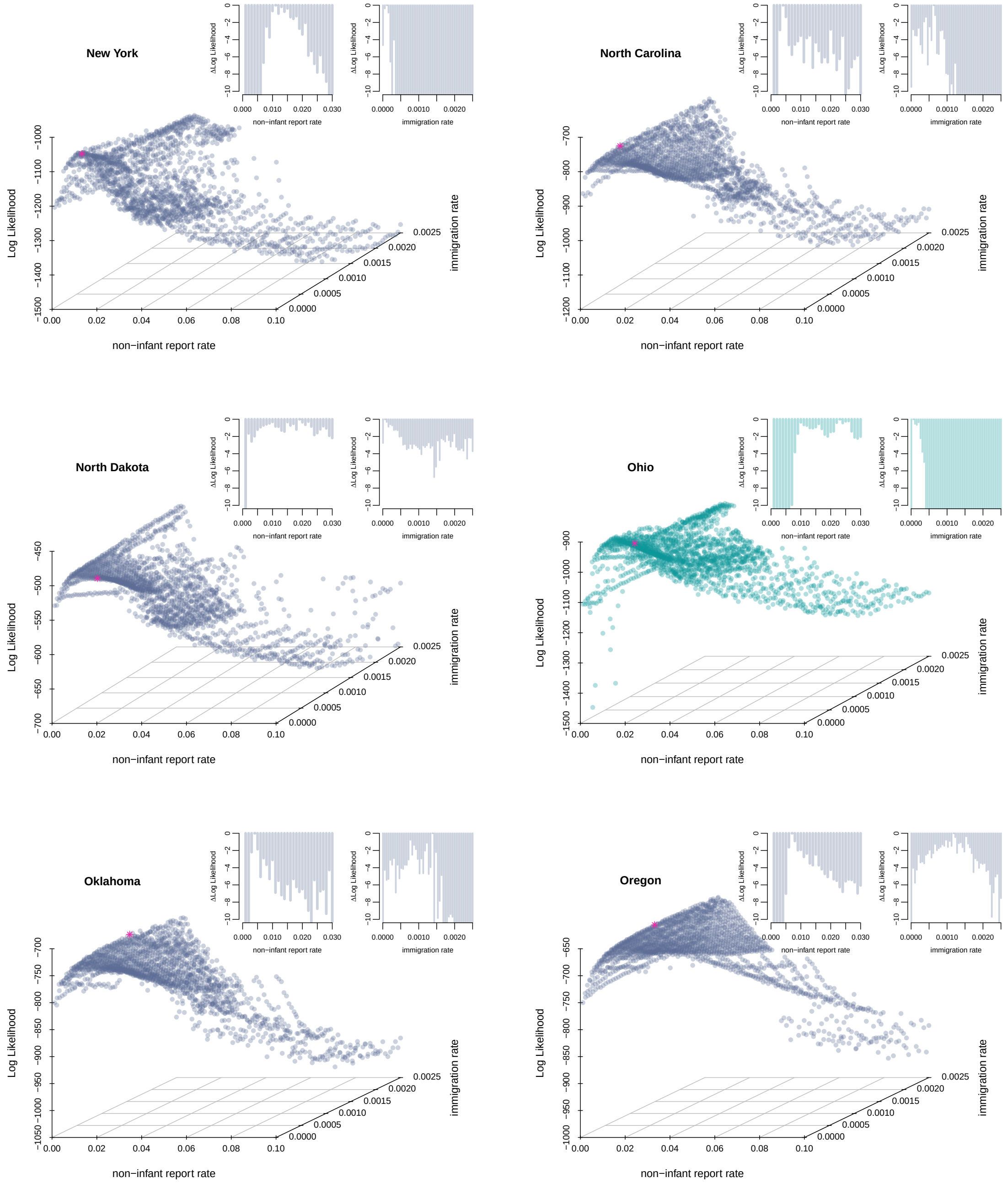

Figure S14: Likelihood profiles for the Pre-Baby Boom non-infant report rate (ρ_t) and the immigration rate (ψ). MLE indicated by pink asterisk. Profile color indicates the whether the report rate was increased during the Baby Boom (purple) or if the report rate was constant through time (green).

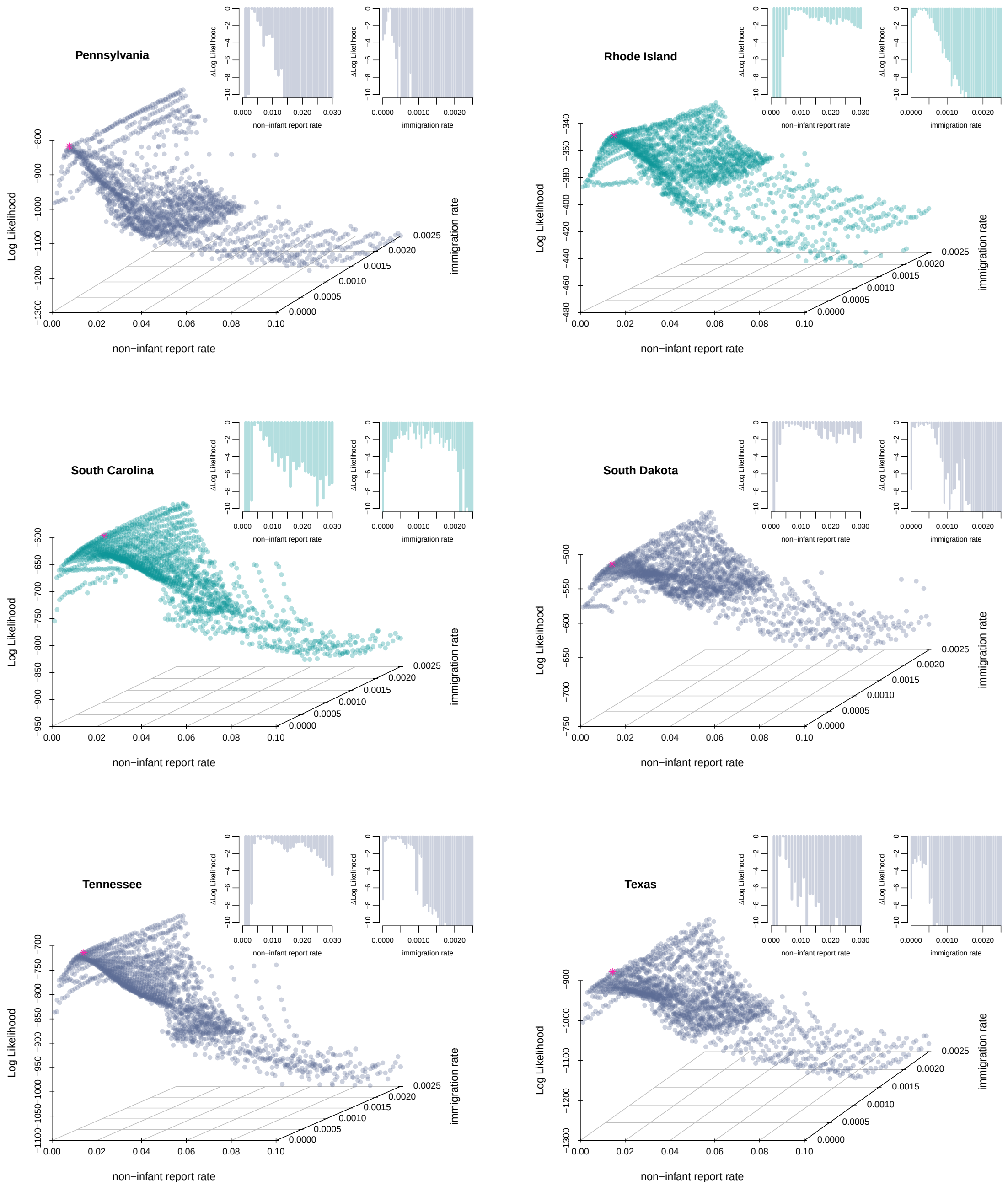

Figure S15: Likelihood profiles for the Pre-Baby Boom non-infant report rate (ρ_t) and the immigration rate (ψ). MLE indicated by pink asterisk. Profile color indicates the whether the report rate was increased during the Baby Boom (purple) or if the report rate was constant through time (green).

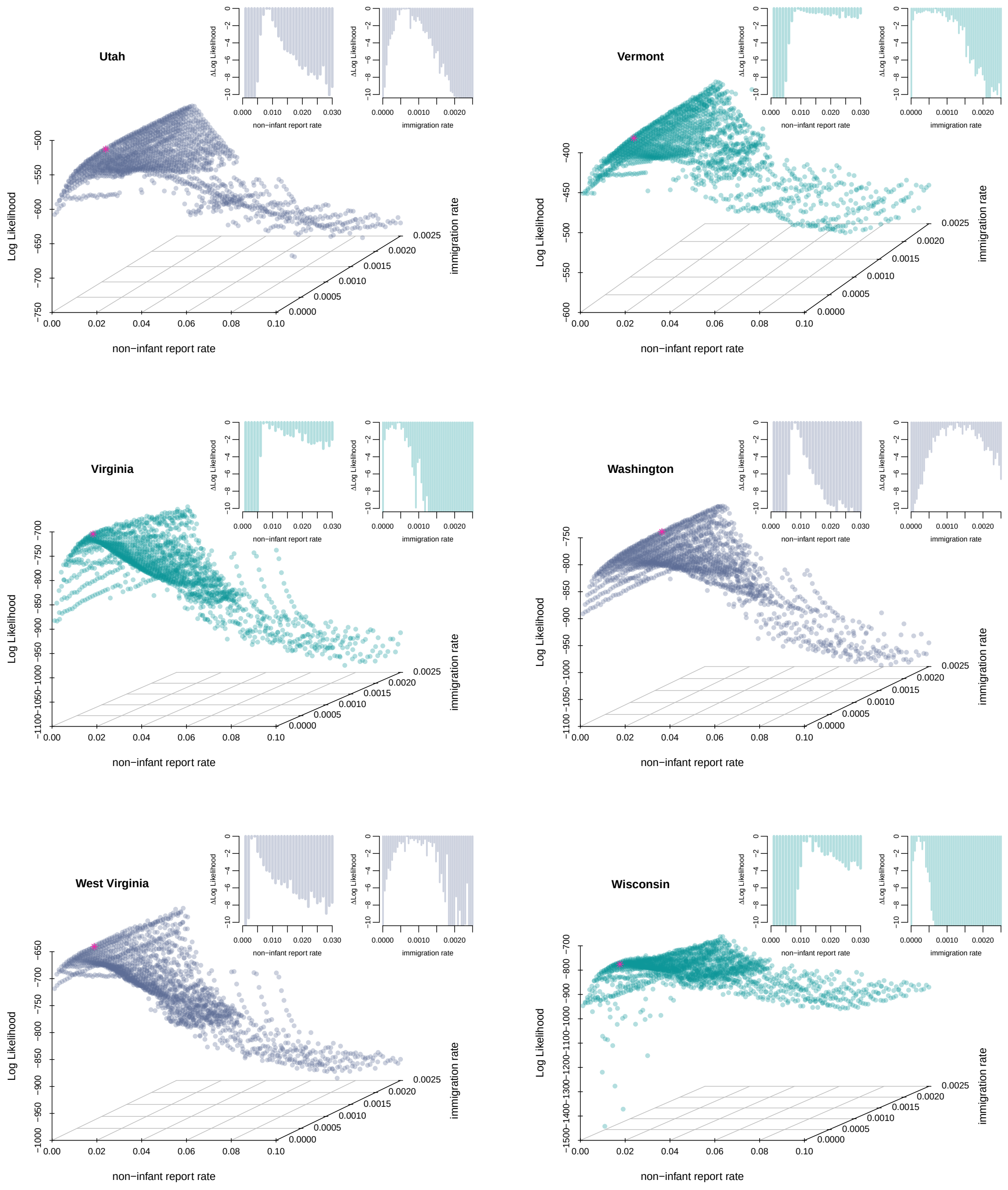

Figure S16: Likelihood profiles for the Pre-Baby Boom non-infant report rate (ρ_t) and the immigration rate (ψ). MLE indicated by pink asterisk. Profile color indicates the whether the report rate was increased during the Baby Boom (purple) or if the report rate was constant through time (green).

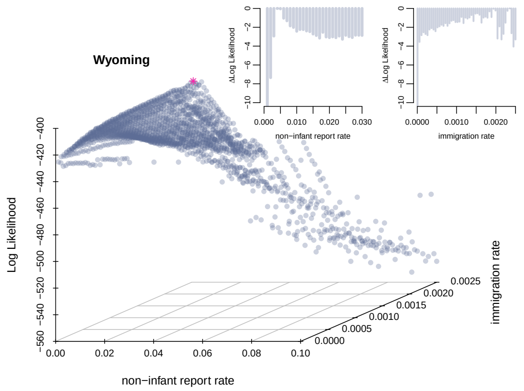

Figure S17: Likelihood profiles for the Pre-Baby Boom non-infant report rate (ρ_t) and the immigration rate (ψ). MLE indicated by pink asterisk. Profile color indicates the whether the report rate was increased during the Baby Boom (purple) or if the report rate was constant through time (green).

References

1. Trevelyan, B, Smallman-Raynor, M, & Cliff, A. D. (2005) The Spatial Dynamics of Poliomyelitis in the United States: From Epidemic Emergence to Vaccine-Induced Retreat, 1910-1971. *Annals of the Association of American Geographers* **95**, 269–293.

2. United States Centers for Disease Control and Prevention. (2010) United States Vital Statistics (http://www.cdc.gov/nchs/products/vsus.htm). Accessed: March 1, 2010.

3. United States Census Bureau. (2010) United States Population Estimates: Historical Estimates (http://www.census.gov/popest/data/historical/index.html). Accessed: March 1, 2010.

4. Bjørnstad, O. N. (2013) Spatial Nonparametric Covariance Function (R package ncf).

5. Gouhier, T. (2014) *biwavelet: Conduct univariate and bivariate wavelet analyses*. (Version 0.17.4).

6. Iannone, R. (2015) DiagrammeR: Create Diagrams and Flowcharts Using R.

7. Alexander, J. P, Gary, H. E, & Pallansch, M. A. (1997) Duration of Poliovirus Excretion and its Implications for Acute Flaccid Paralysis Surveillance: A Review of the Literature. *The Journal of Infectious Diseases* **175, Suppl**, S176–S182.

8. King, A. A, Ionides, E. L, Breto, C. M, Ellner, S, Kendall, B, Wearing, H, Ferrari, M. J, Lavine, M, & Reuman, D. C. (2010) pomp: Statistical Inference for Partially Observed Markov Processes (R package).

9. King, A. A, Nguyen, D, & Ionides, E. (in press) Statistical Inference for Partially Observed Markov Processes via the R Package pomp. *Journal of Statistical Software*.

10. Keeling, M & Rohani, P. (2008) *Modeling Infectious Diseases in Humans and Animals*. (Princeton University Press, Princeton).

11. Martinez-Bakker, M, Bakker, K, King, A. A, & Rohani, P. (2014) Human Birth Seasonality: Latitudinal Gradient and Interplay with Childhood Disease Dynamics. *Proceedings of the Royal Society B: Biological Sciences* **281**.

12. Dorélien, A, Ballesteros, S, & Grenfell, B. T. (2013) Impact of Birth Seasonality on Dynamics of Acute Immunizing Infections in Sub-Saharan Africa. *PLoS ONE* **8**, e75806.
